# Hedgehog pathway dysregulation contributes to the pathogenesis of human gastrointestinal stromal tumors *via* GLI-mediated activation of *KIT* expression

**DOI:** 10.18632/oncotarget.12909

**Published:** 2016-10-25

**Authors:** Chih-Min Tang, Tracy E. Lee, Sabriya A. Syed, Adam M. Burgoyne, Stephanie Y. Leonard, Fei Gao, Jonathan C. Chan, Eileen Shi, Juliann Chmielecki, Deborah Morosini, Kai Wang, Jeffrey S. Ross, Michael L. Kendrick, Michael R. Bardsley, Martina De Siena, Junhao Mao, Olivier Harismendy, Tamas Ordog, Jason K. Sicklick

**Affiliations:** ^1^ Department of Surgery, Division of Surgical Oncology, Moores UCSD Cancer Center, University of California, San Diego, La Jolla, California, USA; ^2^ Department of Physiology and Biomedical Engineering and Gastroenterology Research Unit, Enteric Neuroscience Program, Division of Gastroenterology and Hepatology, Mayo Clinic, Rochester, Minnesota, USA; ^3^ Department of Biochemistry and Molecular Biology, Mayo Clinic, Rochester, Minnesota, USA; ^4^ Department of Medicine, Division of Hematology/Oncology, Moores UCSD Cancer Center, University of California, San Diego, La Jolla, California, USA; ^5^ School of Medicine, University of California, San Diego, La Jolla, California, USA; ^6^ Foundation Medicine, Inc., Cambridge, Massachusetts, USA; ^7^ Department of Surgery, Mayo Clinic, Rochester, Minnesota, USA; ^8^ Department of Molecular, Cell and Cancer Biology, University of Massachusetts, Worchester, Massachusetts, USA; ^9^ Division of Biomedical Informatics, Moores UCSD Cancer Center, University of California San Diego, La Jolla, California, USA; ^10^ Center for Individualized Medicine, Mayo Clinic, Rochester, Minnesota, USA

**Keywords:** arsenic trioxide, GIST, GLI, ICC, imatinib-resistant

## Abstract

Gastrointestinal stromal tumors (GIST) arise within the interstitial cell of Cajal (ICC) lineage due to activating *KIT/PDGFRA* mutations. Both ICC and GIST possess primary cilia (PC), which coordinate PDGFRA and Hedgehog signaling, regulators of gastrointestinal mesenchymal development. Therefore, we hypothesized that Hedgehog signaling may be altered in human GIST and controls *KIT* expression. Quantitative RT-PCR, microarrays, and next generation sequencing were used to describe Hedgehog/PC-related genes in purified human ICC and GIST. Genetic and pharmacologic approaches were employed to investigate the effects of *GLI* manipulation on *KIT* expression and GIST cell viability. We report that Hedgehog pathway and PC components are expressed in ICC and GIST and subject to dysregulation during GIST oncogenesis, irrespective of *KIT/PDGFRA* mutation status. Using genomic profiling, 10.2% of 186 GIST studied had potentially deleterious genomic alterations in 5 Hedgehog-related genes analyzed, including in the *PTCH1* tumor suppressor (1.6%). Expression of the predominantly repressive GLI isoform, *GLI3*, was inversely correlated with *KIT* mRNA levels in GIST cells and non-*KIT*/non-*PDGFRA* mutant GIST. Overexpression of the 83-kDa repressive form of GLI3 or small interfering RNA-mediated knockdown of the activating isoforms GLI1/2 reduced *KIT* mRNA. Treatment with GLI1/2 inhibitors, including arsenic trioxide, significantly increased GLI3 binding to the *KIT* promoter, decreased *KIT* expression, and reduced viability in imatinib-sensitive and imatinib-resistant GIST cells. These data offer new evidence that genes necessary for Hedgehog signaling and PC function in ICC are dysregulated in GIST. Hedgehog signaling activates *KIT* expression irrespective of mutation status, offering a novel approach to treat imatinib-resistant GIST.

## INTRODUCTION

Gastrointestinal stromal tumor (GIST) is the most common sarcoma with an estimated annual incidence of 6.8 cases per million people in the United States [1]. GIST is thought to arise from stem cells that differentiate toward the lineage of interstitial cells of Cajal (ICC), electrical pacemaker and neuromodulator cells of the gut [2–5]. ICC represent ~5% of the cells in the *tunica muscularis* [6] and their development depends upon KIT (v-kit Hardy-Zuckerman 4 feline sarcoma viral oncogene homolog; CD117) receptor tyrosine kinase (RTK) expression and signal transduction. Approximately 95% of GIST express KIT [7]. GIST oncogenesis involves somatic activating alterations in *KIT* (75-80%) or platelet-derived growth factor receptor α (*PDGFRA*; <10%) [2]. In rare cases, GIST lack a mutant *KIT* or *PDGFRA* allele. These tumors may arise from mutations in KIT/PDGFRA signaling intermediates (e.g., *NF1, BRAF, KRAS, HRAS*) or deficiency in the mitochondrial succinate dehydrogenase (SDH) complex caused by inactivating SDH subunit (A-D) mutations or epigenetic repression [2, 3, 8, 9]. Despite their molecular heterogeneity, most GIST share common characteristics, including expression and activation of KIT and PDGFRA. Indeed, depending on the tumor genotype, about half to three quarters of patients with advanced GIST respond to treatment with anti-KIT/PDGFRA tyrosine kinase inhibitors (TKIs) including the first-line drug, imatinib mesylate (Novartis Pharmaceuticals, Switzerland). However, TKIs alone do not eradicate GIST cells, and more than 95% of patients eventually succumb to imatinib-resistant disease [2], necessitating the search for alternative therapeutic targets.

The Hedgehog signaling pathway is critical for the development of the gastrointestinal tract, including the growth of the underlying mesenchyme [10, 11]. Here, Hedgehog activation promotes the survival and proliferation of *tunica muscularis* progenitors [11]. During embryonic development, ectopic Hedgehog pathway activation in the murine pancreatic bud stimulates the differentiation of the surrounding mesoderm into gut-like mesenchyme containing smooth muscle cells and KIT^+^ ICC-like cells [12]. Throughout life, the Hedgehog pathway is regulated by ligand-dependent and/or ligand-independent mechanisms. With the aid of dispatched (DISP), the hedgehog ligands, sonic hedgehog (SHH) and indian hedgehog (IHH), are secreted and then bind to the patched 1 and 2 receptors (PTCH1 and PTCH2). In the absence of hedgehog ligands, PTCH1/2 bind to, and repress the activity of, the smoothened co-receptor (SMO). In turn, the GLI family of transcription factors remains in a balance favoring transcriptional repression, with GLI3 being proteasomally processed into a transcriptional repressor (GLI3R). However, in the presence of SHH/IHH ligand binding to PTCH1/2 and their co-receptors, SMO inhibition is released, leading to transcriptional activation by GLI1 and GLI2, as well as reduced transcriptional repression by GLI3 [13]. Suppressor of fused (SUFU) functions as a tumor suppressor that inhibits the GLI transcription factors and suppresses the Hedgehog pathway [14]. Ultimately, Hedgehog signaling controls the expression of its own intermediates, including PTCH1, as well as genes that control cell proliferation, survival, epithelial-to-mesenchymal transition, stemness, and other developmental pathways [15].

Elevated Hedgehog signaling results from loss-of-function mutations in PTCH1 and SUFU, gain-of-function mutations in SMO, or overexpression of pathway activators including SHH/IHH ligands, SMO or GLI1/2, which can lead to aberrant cell growth and tumorigenesis [16]. However, only a small amount evidence has been accumulated to indicate a role for the Hedgehog pathway in GIST.[17] SHH, PTCH1, SMO, and GLI1 expression were detected by immunohistochemistry in one study [18], and chromosome 7p amplification was found to be associated with increased *GLI3* expression [19]. Interestingly, GLI3 has been reported to repress *KIT* mRNA levels in ICC-like cells of the murine ureter [20], raising the possibility that GLI3 may contribute to the formation of a KIT^low/−^ GIST cell pool [4] responsible, in part, for disease persistence during imatinib therapy [3]. Additionally, conditional PTCH1 inactivation in lysozyme M-expressing murine cells has been reported to lead to the development of PDGFRA^+^ GIST-like lesions [21]. Finally, it is known that optimal Hedgehog signaling in vertebrate cells requires primary cilia (PC) [22]. PC have been reported in murine, rat, rabbit, and human ICC [23, 24], as well as in primary, recurrent and metastatic human GIST [25, 26], possibly developing under the control of the ICC and GIST marker, anoctamin 1 (ANO1) [27].

Here, we tested the hypothesis that the Hedgehog pathway contributes to GIST oncogenesis. We report that Hedgehog-related genes are robustly expressed in isolated human and murine ICC, ICC stem cells, and GIST, irrespective of mutation status. We also detected potentially significant genomic alterations in key Hedgehog pathway members (*PTCH1, PTCH2, SMO, SUFU, GLI1*) in 10% of GIST studied. We also found that GIST oncogenesis is associated with a shift in Hedgehog-related gene expression relative to normal human ICC. Finally, we demonstrate reduced *KIT* expression in response to genetic or pharmacological blockade of GLI1/2 or overexpression of the repressor form of GLI3 and show reduced cell viability in imatinib-sensitive and imatinib-resistant cells in response to pharmacological inhibition of Hedgehog signaling. Together, these results show that the Hedgehog pathway is a potential novel therapeutic target in TKI-resistant GIST.

## RESULTS

### Murine GIST models express Hedgehog signaling components

Given that the Hedgehog pathway has been shown to control the development of the mouse gut mesenchyme [11], we sought to determine whether two commonly studied murine models of the mesenchymal tumor GIST express key Hedgehog signaling components (*Shh, Ihh, Ptch1, Smo, Gli1, Gli2, Gli3*). We studied *Kit*^V558Δ/+^ [28] and *Kit*^+/K641E^ transgenic mice [29]. Total RNA was extracted from murine cecal GIST (*n* = 3 mice/model) and quantitative RT-PCR analyses were performed (Supplementary Figure S1A). Kit served as positive control and *Actb* served as loading control. Both models highly expressed several canonical Hedgehog pathway components, including *Ihh, Ptch1, Smo, Gli1* and *Gli2*. There was little or no *Shh* or *Gli3* expression in either model. We confirmed our findings by gel electrophoresis of the PCR products (Supplementary Figure S1B). These findings demonstrate that two murine models of GIST express several key Hedgehog signaling components.

### Human GIST cell lines express Hedgehog signaling components

**Figure 1:**
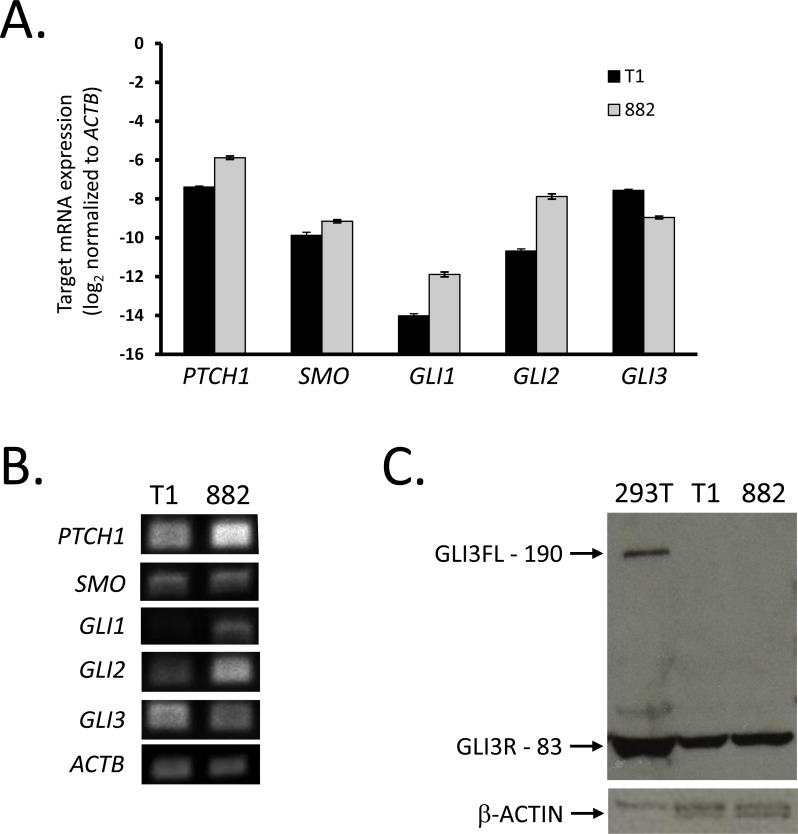
GIST cell lines express Hedgehog signaling components **A.** Following RNA extraction from GIST-T1 and GIST882, real-time RT-PCR analysis was performed for Hedgehog signaling components using the ΔCt method and *ACTB* as reference. Both cell lines were studied in three independent experiments, and each experiment was performed in triplicate. **B.** Agarose gel electrophoresis of two-step RT-PCR products shows expression of Hedgehog signaling components. **C.** Western blot analyses performed in whole cell lysates from GIST-T1, GIST882, and HEK293T cells (positive control) showing full length (FL, 190 kDa) or repressor (R, 83 kDa) isoforms of GLI3. β-actin served as loading control.

To determine if Hedgehog pathway mRNA expression is present in human GIST, we first studied two human gastric GIST cell lines, GIST-T1 and GIST882. Similarly to the *Kit*^V558Δ/+^ mice, GIST-T1 has an exon 11 mutation (*KIT* V560-Y579Δ5), and GIST882 cells bear the same *KIT* mutation as the *Kit*^+/K641E^ transgenic mice (K642E; exon 13). Using quantitative RT-PCR analyses, we evaluated the expression of the seven aforementioned Hedgehog pathway components (Figure 1A). While both cell lines lack expression of *SHH* and *IHH* (data not shown), we confirmed that the lines express high levels of *PTCH1, SMO, GLI2*, and *GLI3. GIST882* had 3.3-fold higher *PTCH1*, 2.0-fold higher *SMO*, and 6.0-fold higher *GLI2* mRNA levels, as well as 2.2-fold lower *GLI3* mRNA expression relative to GIST-T1 (Figure 1A). Thus, GIST-T1 more robustly expresses *GLI3*, while GIST882 more robustly expresses *GLI2*. We confirmed our findings by gel electrophoresis of the PCR products (Figure 1B). To assess whether the GLI3 protein was full length (GLI3FL, 190 kDa) or proteolytically processed into the shorter GLI3 repressor (GLI3R, 83 kDa), we performed Western blot analysis with HEK293T cells as a positive control for expression of both GLI3 isoforms. We found both GIST-T1 and GIST882 to only express GLI3R without detectable GLI3FL (Figure 1C). These human gastric cell lines appeared to have lower *IHH* and *GLI1* mRNA, but higher *GLI3* mRNA levels than the murine cecal tumors, suggesting that these models have different Hedgehog pathway expression patterns.

### Human GIST express Hedgehog signaling components

**Figure 2:**
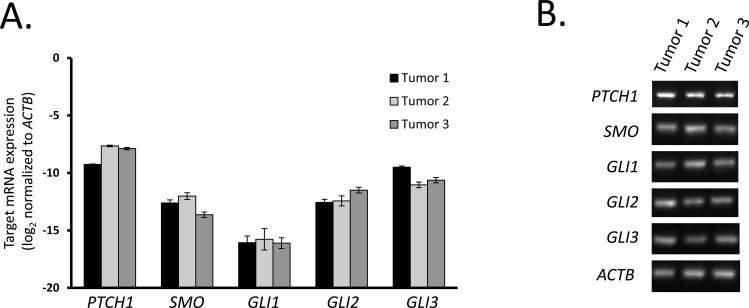
Tumor tissues from human GIST express Hedgehog signaling components **A.** Following RNA extraction from 3 human GIST tumors, real-time RT-PCR analysis was performed for Hedgehog signaling components. mRNA expression was quantified by the ΔCt method using *ACTB* as reference. **B.** Agarose gel electrophoresis of two-step RT-PCR products shows expression of Hedgehog signaling components.

We next sought to confirm the expression of Hedgehog pathway components in freshly excised human tumors. Using the same human RT-PCR primers, we evaluated the Hedgehog pathway expression in three small bowel GIST (duodenal, jejunal, and ileal) from patients with *KIT* exon 9, 11, and 9 mutations, respectively. The human tumor data paralleled our cell line findings in that there was high *PTCH1, SMO, GLI2*, and *GLI3* mRNA, but low *GLI1* mRNA (Figure 2A-2B). Moreover, *SHH* and *IHH* mRNA expression was not detectable by quantitative RT-PCR analyses (data not shown). Therefore, the gastric GIST cell lines faithfully represent Hedgehog expression patterns in the human small intestinal tumors and the differences from mouse GIST appear to reflect species differences rather than differences in anatomical location or phenotypic changes during culturing.

### The Hedgehog pathway is inherent to the ICC lineage

To investigate whether expression of the Hedgehog pathway occurs only after GIST oncogenesis or is inherent to the ICC lineage (i.e. the histogenetic source of GIST), we purified ICC by FACS from the stomach of patients undergoing bariatric surgery (*n* = 6). Gene expression was determined by Affymetrix microarrays and compared to dissociated, unfractionated gastric *tunica muscularis* samples (*n* = 4). By subsequent MetaCore™ analysis, unique genes significantly increased in ICC vs. their source tissue (log_2_ fold change >1, Benjamini-Hochberg *Q*<0.05) were significantly enriched in genes assigned to the “Development_Hedgehog Signaling” network, which ranked 29^th^ among biological process networks (Supplementary Table S1). This pathway was also significantly represented in gene sets differentially expressed in FACS-purified mouse small intestinal ICC-MY and ICC-DMP [30], as well as in the mouse gastric ICC-SC line 2xSCS2F10 [31] relative to their own source tissues. These results indicate that the Hedgehog pathway is intrinsic to the cells of the ICC lineage in both mice and humans.

### The Hedgehog pathway is altered during GIST oncogenesis

**Figure 3:**
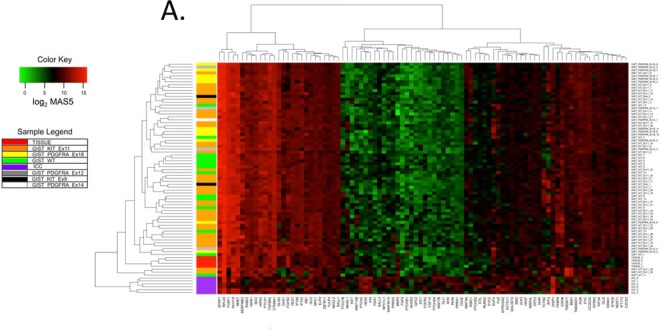

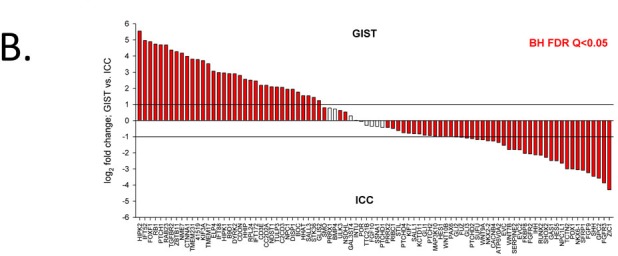
Hedgehog signaling-related genes are differentially expressed by human gastric GIST, purified human ICC and their source tissue by microarray analysis The list of Hedgehog-signaling-related genes was assembled by searching the Affymetrix Human Genome U133 Plus 2.0 microarray annotation file (na33) for Gene Ontology (GO) terms containing “smoothened” (biological process) and “hedgehog” (molecular function) (see specific GO terms in http://www.ebi.ac.uk/GOA). **A.** Heat map and unsupervised hierarchical cluster analysis of 69 human gastric GIST of various genotypes from the NCBI GEO series GSE17743 [32], GSE8167 [33], and GSE20708 [19] (see Supplementary Table S2 for details), FACS-sorted human ICC (*n* = 6), and unfractionated gastric *tunica muscularis* tissues (Tissue; *n* = 4). Expression values were determined using the MAS5 statistical algorithm. Significance of expression was determined by Wilcoxon signed-rank test. Heatmaps were assembled using probe set expression values with the lowest *P* value. Red and green colors specify high and low expression, respectively. Mutation status of each human GIST is indicated (WT: wild-type as defined in the referenced studies). Cluster analysis of Hedgehog-related genes differentiated GIST from ICC but not GIST with different genotypes. **B.** Differential expression of Hedgehog signaling-related genes detected by microarray analysis in human gastric *KIT*-mutant GIST from the NCBI GEO series GSE17743 [32] (*n* = 15) and FACS-purified human ICC (*n* = 6). Expression values were determined by RMA. Unique gene lists were created by identifying the probe sets with the lowest Benjamini-Hochberg false discovery rate (BH FDR) *Q* values. Horizontal lines indicate threshold for significant log_2_ fold changes; red fill indicates *Q* < 0.05 from BH FDR. Differential expression with log_2_ fold change < −1 or >1 and *Q* < 0.05 was considered significant. Thirty-five Hedgehog-related genes were significantly overexpressed in GIST including Hedgehog signaling pathway and transcriptional target genes, as well as genes encoding for proteins responsible for anterograde cargo transport in PC. Notably, the 29 genes significantly overexpressed in ICC included the Hedgehog ligands *SHH* and *IHH*.

We next performed heat map and unsupervised hierarchical cluster analysis to investigate whether Hedgehog pathway expression patterns change during GIST oncogenesis. MAS5 expression values of unique genes in human gastric ICC (*n* = 6), unfractionated gastric *tunica muscularis* tissue (*n* = 4) and human gastric GIST microarrays (*n* = 69; Supplementary Table S2 [19, 32, 33]) and assigned to the Gene Ontology (GO) terms containing “smoothened” (biological process) and “hedgehog” (molecular function) were analyzed (Figure 3A). Cluster analysis clearly differentiated ICC from GIST. In contrast, GIST with different driver mutations (KIT exon 9 and 11, *PDGFRA* exon 12, 14 and 18, as well as GIST lacking both *KIT* and *PDGFRA* mutations) could not be distinguished by their Hedgehog pathway expression patterns. Similarly to the results obtained by RT-PCR in small intestinal *GIST, PTCH1, GLI3* and *GLI2* but not *IHH* and *SHH* were expressed in gastric GIST. *SMO* was also expressed, albeit at lower levels.

We also performed pairwise analyses of GIST, ICC, and unsorted gastric *tunica muscularis* focusing on the same Hedgehog- and SMO-related gene set (Figure 3B and Supplementary Figure S2A-B). Thirty-five Hedgehog-related genes were more than 2-fold overexpressed in GIST as compared to ICC. These included pathway signaling components such as *PTCH1, RAB23, CDON, HHIP, DISP1*, and *BOC*. It is noteworthy that two of these are Hedgehog target genes, namely, *PTCH1* and *HHIP*, function as readouts of pathway activity, and act to fine-tune Hedgehog signals based upon negative feedback loops. Given the reported presence of primary cilia (PC) in GIST [25, 26], as well as the importance of PC in Hedgehog signaling transduction, it is also noteworthy that four proteins responsible for anterograde trafficking of cargo in PC, namely, *KIF3A* (encoding kinesin family member 3A), *IFT52* (intraflagellar transport 52), IFT88 (intraflagellar transport 88), and *IFT172* (intraflagellar transport 172), were significantly overexpressed in GIST as compared to ICC (Figure 3B). Overall, the top 7 overexpressed (>16-fold) genes in GIST relative to ICC (in descending order) are *HIPK2* (homeodomain-interacting protein kinase 2), *IFT52, FOXF1* (forkhead box F1), *RB1* (retinoblastoma 1), *PtTCH1, RAB23*, and *TGFBR2* (transforming growth factor beta receptor II). In contrast, 29 genes were more than 2-fold decreased in GIST as compared to ICC, including *SHH, IHH, GAS1, FKBP8, SUFU*, and *GLI3*. Overall, the 7 most under-expressed (>8-fold) genes in GIST relative to ICC were ZIC1 (Zic family member 1), *FGFR3* (fibroblast growth factor receptor 3), GPC2 (glypican 2), *SHH, FGF9* (fibroblast growth factor 9), *SFRP1* (secreted frizzled-related protein 1), and *NKX6-1* (NK6 homeobox 1). Taken together, these findings corroborate our earlier quantitative RT-PCR analyses demonstrating that Hedgehog-related genes are expressed in GIST, and indicate that many critical signaling components, including those of PC, are increased during the transformation from ICC to GIST regardless of the specific mutation driving oncogenesis.

### Human GIST possess genomic alterations in Hedgehog signaling components

In addition to transcriptional alterations, GIST may also contain potentially pathogenic mutations in Hedgehog pathway members. To investigate this possibility, we performed comprehensive genomic profiling using the FoundationOne^®^ next-generation sequencing assay panel in a separate cohort of 191 GIST from 186 patients. In this cohort, 19 of 186 patients' tumors (10.2%) possessed genomic alterations in 5 Hedgehog-related genes analyzed (i.e., *PTCH1, PTCH2, SMO, SUFU*, and *GLI1*) (Table 1). The average age at diagnosis of this GIST subset was 54.3 ± 19.4 (median 58 years old) with male predominance (male: 63.2%; female: 36.8%). Three GIST (1.6%) of the entire cohort had likely deleterious genomic alterations in *PTCH1*. Of these three tumors, two also had *KIT* exon 11 mutations, and one had an *NF1* mutation. The remaining GIST had “variants of unknown significance” (VUSes) in all 5 genes, which were predicted to be deleterious by at least 2 out of 4 prediction tools (SIFT, PolyPhen, MutationTaster, and/or MutationAssessor). These variants included 7 in *PTCH1*, 3 in *PTCH2*, 4 in *SMO*, 2 in *SUFU*, and 6 in *GLI1*. In addition, these 19 Hedgehog-altered tumors had known oncogenic mutations in *KIT* (*n* = 13, 68.4%), *PDGFRA* (*n* = 1, 5.3%), NF1 (*n* = 1, 5.3%), and SDHA (*n* = 1, 5.3%). Three tumors (15.8%) had no known driver mutations in *KIT, PDGFRA, NF1, KRAS, HRAS*, or *SDHx* subunits. These so-called “wild-type” (WT) GIST had predicted deleterious variants in *SUFU* and *GLI1*. Taken together, somatic mutations in a subset of Hedgehog pathway genes were found in about 10% of GIST that we analyzed, raising the possibility that these genes may act as modifier genes in the development and progression of GIST.

**Table 1:** Genomic alterations in the Hedgehog pathways genes detected in 19 of 186 GIST patients by the FoundationOne^®^ assays

Patient	Hh Pathway Gene	Alteration	Foundation Medicine Category	Allelic Frequency	Genomic Alteration	Depth of NGS Coverage	GIST Driver Gene (s)	KIT/PDGFRΑ Exon(s)	GIST Driver Gene Alteration(s)
**1**	*PTCH1*	E44G	VUS	0.88	missense	478	*KIT*	11	W557_V559>C
**1**	*PTCH1*	S827G	VUS	0.89	missense	714	*KIT*	11	W557_V559>C
**2**	*PTCH1*	E44G	VUS	0.85	missense	506	*KIT*	11, 17	K550_K558del, D820Y
**3**	*PTCH1*	R1239Q	VUS	0.51	missense	480	*SDHA*	-	R171H, R451C
**3**	*PTCH1*	E44del	VUS	0.33	inframe indel	621	*SDHA*	-	R171H, R451C
**4**	*PTCH1*	V1418I	VUS	0.51	missense	349	*KIT*	11, 13	W557_K558del, V654A
**5**	*PTCH1*	W1339_R1345del	likely deleterious	0.16	inframe indel	535	*KIT*	11	W557_K558del
**5**	*PTCH1*	E44G	VUS	0.51	missense	359	*KIT*	11	W557_K558del
**6**	*PTCH1*	PTCH1_C9orf153_truncation	likely deleterious		truncation		*NF1*	-	V1146I
**7**	*PTCH1*	S181*	likely deleterious	0.42	nonsense	553	*KIT*	11	V560D
**8**	*PTCH2*	S1189T	VUS	0.61	missense	345	*KIT*	11, 14	W557_K558del, T670I
**9**	*PTCH2*	A531V	VUS	0.46	missense	362	*KIT*	11, 13	W557_K558>Q, V654A
**7**	*PTCH2*	P881Q	VUS	0.11	missense	338	*KIT*	11	V560D
**10**	*SMO*	G16_L17insL	VUS	0.21	inframe indel	439	*KIT*	9	S501_A502insAY
**11**	*SMO*	R671W	VUS	0.51	missense	414	*KIT*	11	V560E
**12**	*SMO*	R726Q	VUS	0.89	missense	310	*KIT*	11, 17	V560_E561>E, D820V, Y823D
**13**	*SMO*	V54M	VUS	0.53	missense	131	*PDGFRΑ*	18	D842V
**14**	*SUFU*	A425V	VUS	0.05	missense	618	*WT*	-	
**15**	*SUFU*	R362C	VUS	0.5	missense	479	*WT*	-	
**16**	*GLI1*	R293C	VUS	0.46	missense	428	*WT*	-	
**15**	*GLI1*	A745V	VUS	0.49	missense	475	*WT*	-	
**17**	*GLI1*	splice site 913-1G>A	VUS	0.38	splicing	519	*KIT*	11	W557_K558del
**18**	*GLI1*	G274C	VUS	0.52	missense	923	*KIT*	11	D579_H580insDPTQLPYD
**19**	*GLI1*	P666S	VUS	0.48	missense	705	*KIT*	9	Y503_F504insAY
**19**	*GLI1*	G421S	VUS	0.48	missense	688	*KIT*	9	Y503_F504insAY

Abbreviations: NGS, next-generation sequencing; VUS, variant of unknown significance; WT, wild type tumor (lacking mutations in KIT, PDGFRA, BRAF, KRAS, HRAS, NF1, and SDH subunits).

### KIT expression inversely correlates with GLI3 expression

**Figure 4:**
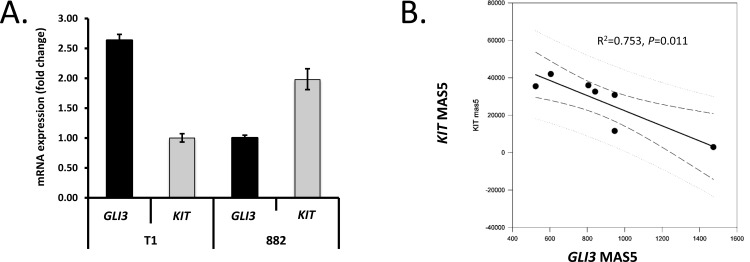

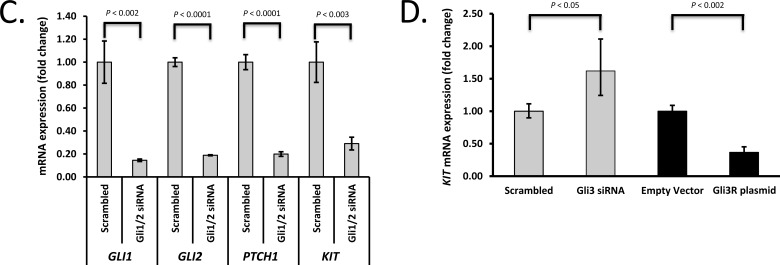

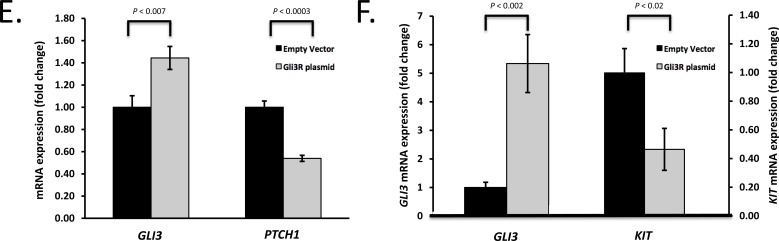
Genetic modulation of GLI transcription factors controls KIT expression **A.** Using quantitative RT-PCR, *GLI3* and *KIT* expression were analyzed in two GIST cell lines (*n* = 3 independent experiments). Note inverse relationship between *GLI3* and *KIT* mRNA in GIST-T1 and GIST882 cells. **B.** *KIT* mRNA expression is inversely correlated with *GLI3* mRNA expression in the wild-type (WT) subset (*n* = 7) of GIST microarrays in the NCBI GEO series GSE17743 [32] and GSE20708 [19]. Results from linear regression (solid line) and Pearson product moment correlation are shown. Dashed and dotted lines represent 95% confidence intervals for the regression and the population, respectively. **C.** Genetic inhibition of *GLI1* and *GLI2* reduces expression of *PTCH1* and *KIT* in GIST cells. GIST882 cells were co-transfected with *GLI1* and *GLI2* siRNA or corresponding scrambled sequences (*n* = 3) and analyzed for *GLI1*, *GLI2*, *PTCH1* and *KIT* expression by quantitative RT-PCR 48 h later. **D.** GLI3 moderates *KIT* expression in KIT^+^ GIST cells. GIST882 cells were electroporated with *GLI3* siRNA, corresponding scrambled sequence, GLI3R plasmid or empty vector. At 48 h, GLI3 was successfully knocked down with resultant increased *KIT* mRNA expression as compared to scrambled siRNA (*n* = 3). In contrast, in GIST882 cells electroporated with GLI3R plasmid, *KIT* mRNA decreased at 48 h relative to empty vector (*n* = 3). **E.** GLI3R inhibits *PTCH1* expression in GIST cells. GIST882 cells were transfected with GLI3R plasmid or empty vector and *GLI3* and *PTCH1* mRNA expression was analyzed at 48 h. F. GLI3R inhibits *KIT* expression in imatinib-resistant, KIT^+^ GIST cells. GIST48IM cells were transfected with GLI3R plasmid or empty vector and *GLI3* and *KIT* mRNA expression was analyzed at 48 h. *P*-values are indicated in the panels.

Having identified that the Hedgehog pathway is genomically and transcriptionally dysregulated in the progression from ICC to GIST, we investigated how this pathway may intersect with known oncogenic drivers of GIST. An earlier report by Cain and colleagues demonstrated that GLI3 represses the expression of *KIT* mRNA in murine ureteral ICC-like cells [20]. To determine if GLI3 could be negatively regulating *KIT* mRNA expression in GIST, we further analyzed the quantitative RT-PCR data generated in the GIST cell lines (*n* = 3 independent experiments). This analysis showed an inverse relationship between *GLI3* and *KIT* mRNA levels as the GIST-T1 cell line expressed less *KIT* and more *GLI3* (ratio: 0.38) than the GIST882 line, which expressed *KIT* and *GLI3* at a ratio of 1.98 (Figure 4A).

To extend the validity of this finding to GIST tumors, we analyzed *KIT* expression in relation to *GLI1*, *GLI2* and *GLI3* expression in 46 gastric GIST microarrays from GSE17743 [32] and GSE20708 [19]. We found no significant correlation between the expression of *KIT* and any of the *GLI* genes in the entire combined dataset. However, in WT GIST (defined as GIST lacking *KIT* and *PDGFRA* mutations; *n* = 7), KIT and *GLI3* expression were inversely correlated (*R*^2^ = 0.753, *P* = 0.011; Figure 4B). Taken together, expression of the Hedgehog transcription factor, *GLI3*, inversely correlates with *KIT* mRNA levels in GIST cells (Figure 4A) and in non-*KIT*/non-*PDGFRA* mutant GIST tumors (Figure 4B).

### Genetic modulation of GLI transcription factors controls expression of Hedgehog target genes

Using small interfering RNAs (siRNAs) and gene overexpression, we next investigated the effects of modulating *GLI1, GLI2*, and *GLI3* on the expression of the known Hedgehog target gene, *PTCH1*, and *KIT*. Due to GIST-T1 cells' resistance to electroporation, we focused on the GIST882 cell line. Forty-eight hours following introduction of siRNAs targeting *GLI1* or *GLI2* or corresponding non-targeting (scrambled) sequences by nucleofection, *GLI1* and *GLI2* mRNA was reduced by 76% (*n* = 3, *P* = 0.0049) and 38% (*n* = 3, *P* = 0.24; not shown), respectively. *KIT* mRNA was only variably or marginally reduced by *GLI1* knockdown (55%, *n* = 3, *P* = 0.08) or *GLI2* knockdown (19%, *n* = 3, *P* = 0.15; not shown). Given the overlapping functions of GLI1 and GLI2, we assessed the effect of simultaneous knockdown of both genes 48 hours after introduction of siRNAs targeting *GLI1* and *GLI2* as compared to non-targeting sequences. In combination, *GLI1* and *GLI2* siRNAs reduced *GLI1* expression by 84% (*P* = 0.0013) and *GLI2* expression by 81% (*P* < 0.0001). Concomitantly, *PTCH1* mRNA decreased by 80% (*P* < 0.0001) and *KIT* mRNA decreased by 71% (*P* = 0.0027) (Figure 4C). Thus, the two activating forms of GLI positively regulate the expression of the Hedgehog target gene, *PTCH1*, as well as the putative Hedgehog target gene, *KIT*, in GIST882.

We next assessed the role of the Hedgehog transcriptional repressor, *GLI3*, in *KIT* transcriptional regulation. In GIST882 cells, a 10% reduction of *GLI3* mRNA (*n* = 3, *P* = 0.16) was associated with a 62% (*P* = 0.04) increase in *KIT* mRNA expression (Figure 4D). Conversely, inducing *GLI3* overexpression by transfection with a *GLI3R* plasmid (repressor form; *n* = 3; control: empty vector) resulted in a 63% decrease (*P* = 0.001) in *KIT* mRNA expression (Figure 4D). We also examined whether the established Hedgehog target gene *PTCH1* was similarly subject to transcriptional downregulation by GLI3 in GIST882 cells. Indeed, a 44% increase in *GLI3R* (*P* = 0.0064) by *GLI3R* overexpression resulted in a 46% decrease (*P* = 0.0002) in *PTCH1* mRNA levels (Figure 4E). The effect of *GLI3R* overexpression was then assessed in the imatinib-resistant GIST48IM line possessing *KIT* exon 11 and 17 mutations. Compared to empty vector, a 5.3-fold *GLI3R* overexpression (*P* = 0.0019) resulted in a 54% decrease (*P* = 0.015) in *KIT* mRNA expression (Figure 4F), but only an 8% decrease in *PTCH1* mRNA expression (not shown). To determine if the GLI3 transcription factor directly or indirectly regulates *KIT* mRNA expression, we performed GLI3 chromatin immunoprecipitation (ChIP) assays. Both GIST-T1 and GIST882 cells were treated with the GLI1/2 inhibitor, arsenic trioxide (ATO; 4 μM) [34, 35] or vehicle control for 48 hours. Chromatin fragments were pulled down by anti-GLI3 antibodies and PCR-amplified to analyze GLI3 binding to the *KIT* promoter (Supplementary Figure S3). We detected GLI3 binding to regions covering and immediately flanking the *KIT* transcription start site which was increased by ATO, especially in the region immediately upstream of the transcription start site in GIST882 cells, where a 2.5-fold (*P* = 0.02) greater occupancy was found in the presence of GLI1/2 blockade. Taken together, similarly to blocking the activating GLI isoforms (i.e., GLI1/2), increasing the repressor form of GLI (i.e., GLI3R) inhibits *KIT* expression, while blocking *GLI3* mRNA increases *KIT* expression. GLI3R-mediated transcriptional downregulation of *KIT* is via direct promoter binding and is preserved in imatinib-resistant GIST cells. These findings are consistent with our gene expression data and an earlier report in mouse ureteral ICC-like cells [20].

### Pharmacologic GLI1/2 inhibition reduces KIT expression

**Figure 5:**
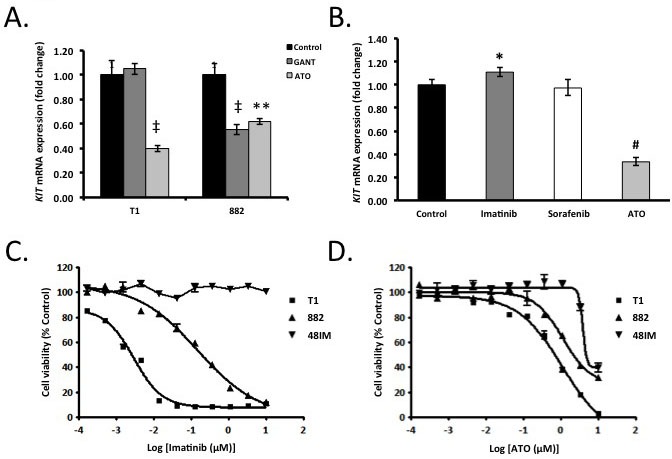
Pharmacologic modulation of Gli transcription factors decreases KIT expression and cell viability **A.** GIST-T1 and GIST882 cells were treated for 48h with 10 μM GANT61 or 4 μM arsenic trioxide (ATO). By qRT-PCR, GLI1/2 pan-inhibition reduced *KIT* mRNA expression versus 0.1% DMSO control in GIST-T1 (ATO only) and GIST882 cells (both GANT61 and ATO). ^‡^ *P* < 0.002. ** *P* < 0.005; *n* = 3/cell line/treatment/group. **B.** Imatinib- and sorafenib-resistant GIST48IM cells were treated with imatinib (10 μM), sorafenib (10 μM) or ATO (4 μM) for 48 h. By qRT-PCR, ATO but not imatinib or sorafenib inhibited *KIT* mRNA expression versus 0.1% DMSO control. * *P* < 0.05, ^#^ *P* < 0.0002; *n* = 3/group. **C.**-**D.** Eleven-point dose-response curves in GIST-T1, GIST882 and GIST48IM cells treated with 10 μM-169 nM imatinib (C) or ATO (D) for 72, 120 and 120 h, respectively. Cell viability was assessed by MTT assay. ATO dose-dependently inhibited GIST-T1, GIST882 and GIST48IM cell viability.

We next utilized the GLI1/2 inhibitor GANT61 (Gli-ANTagonist 61; 10 μM) [36] to simultaneously inhibit both activating GLI transcription factors. GANT61 reduced *KIT* mRNA expression by 50% (*P* = 0.035) in GIST882 cells but not in GIST-T1 cells (*n* = 3/cell line/group, Figure 5A). In contrast, the aforementioned GLI1/2 inhibitor, arsenic trioxide (ATO; 4 μM) [34, 35], which is an FDA-approved drug for the treatment of acute promyelocytic leukemia, reduced KIT mRNA by 60.2% in GIST-T1 cells and 38.0% in the GIST882 line (*n* = 3/cell line/group; Figure 5A). In the imatinib-resistant line GIST48IM, ATO (4 μM) decreased *KIT* mRNA expression by 66.6%, (*P* = 0.0001), whereas imatinib (10 μM) and sorafenib (10 μM) had no effect (Figure 5B). Thus, simultaneous pharmacologic inhibition of GLI1 and GLI2 decreased *KIT* mRNA expression in both imatinib-sensitive and imatinib-resistant GIST [34, 35], similarly to genetic silencing of *GLI1* and *GLI2* in GIST882 cells (Figure 4C).

### Pharmacologic GLI1/2 inhibition reduces GIST cell viability

Suppression of *KIT* mRNA or pharmacological inhibition of KIT signaling in GIST cells is sufficient to induce apoptosis and tumor regression [37]. However, imatinib-resistance due to secondary mutations in *KIT* has emerged as a major clinical problem [2]. In order to exploit the GIST lines' dependence on constitutive *KIT* expression irrespective of *KIT* mutation status, we performed MTT viability assays and 11-point dose response curves with imatinib (positive control) or ATO using both imatinib-sensitive (GIST-T1 and GIST882) and imatinib-resistant (GIST48IM) cells (*n* = 3/cell line/drug/concentration). While both GIST-T1 and GIST882 were sensitive to imatinib (IC_50_ = 2.5 nM and 212 nM, respectively), GIST48IM was resistant to imatinib (IC_50_ > 10 μM) (Figure 5C). On the other hand, all three lines were sensitive to ATO, irrespective of mutation status (Figure 5D). The IC_50_ values were 0.6 μM, 2.7 μM, and 8.8 μM, respectively. These represent clinically achievable concentrations [38]. To assess this effect genetically, we simultaneously knocked down *GLI1* and *GLI2* expression. This alone had minimal effects on cell viability (data not shown), suggesting that the ATO-induced inhibition of *KIT* mRNA may not have sufficiently reduced KIT protein and KIT signaling within the time period of the experiments. However, pharmacologically targeting Hedgehog/GLI signaling, decreasing *KIT* expression, and likely hitting Hedgehog-independent ATO targets (e.g., reactive oxygen species, phosphatase inhibition or JNK/AP-1 activation of redox-sensitive enzymes [39]) in GIST represents a novel approach to disrupting GIST dependence upon KIT signaling for survival, irrespective of imatinib-sensitive or imatinib-resistant *KIT* mutation status.

## DISCUSSION

We provide evidence that the Hedgehog pathway is inherent to the human ICC lineage and altered during GIST oncogenesis. Relative to freshly purified human ICC, tumor cells lose expression of both Hedgehog ligands while increasing expression of genes associated with primary ciliogenesis and Hedgehog pathway target genes, indicating ligand-independent signaling. These transcriptomic alterations occur irrespective of mutation status or anatomic location suggesting that Hedgehog is a conserved signaling pathway in GIST. We also report Hedgehog-related genomic alterations in up to 10% of human GIST, including deleterious mutations in the Hedgehog pathway inhibitor *PTCH1* occurring at a rate of 1.6% in tumors. Using transcriptomic, genetic and pharmacologic approaches, we also demonstrate that the Hedgehog pathway predominantly activates *KIT* expression in human GIST and may be a cell viability factor in both imatinib-sensitive and imatinib-resistant GIST cell lines. Consistent with these data, we observed inhibition of *KIT* mRNA expression and reduced viability in response to ATO (an FDA-approved drug with multiple effects, including GLI1/2 inhibition) in GIST cell lines with different *KIT* mutations, including GIST cells with *KIT* mutation that renders GIST resistant to TKI therapies. Importantly, the ATO doses resulting in these effects appear to be therapeutically relevant [38]. Together, our findings show that the Hedgehog pathway is expressed in ICC and plays a fundamental role in the pathobiology of GIST. Inhibiting Hedgehog/GLI signaling and downstream *KIT* expression, as well as other ATO targets, represents a novel and clinically potentially realizable approach to disrupting GIST dependence on KIT signaling for survival. This is especially important in imatinib-resistant tumors, which represent the most important challenge in the treatment of advanced GIST [2].

Our current findings significantly expand upon earlier reports demonstrating a relationship between GIST and the Hedgehog pathway. In 31 gastric GIST, one study reported expression of SHH (in 58.1% of tumors), PTCH1 (in 77.4%), SMO (in 80.6%), and GLI1 (in 58.1%) by immunohistochemistry [18]. There was strong correlation between SHH protein and risk of GIST recurrence. Contrary to this study, in two GIST cell lines and 72 human gastric and small intestinal GIST, we found low or undetectable expression of Hedgehog ligands and *GLI1*. The difference in these findings may lie in technical factors, as the antibodies employed in ref. [18] are not recommended for use in FFPE sections by the manufacturer. More recently, Pelczar *et al.* suggested a relationship between GIST and the Hedgehog pathway by describing PDGFRA^+^ “GIST-like tumors” in mice with conditional *Ptch1* inactivation in lysozyme M-expressing cells [21]. However, the cells-of-origin could not be identified and, together with the GIST-like tumors, lacked KIT expression, complicating interpretation. In contrast, we find that KIT-expressing human GIST cell lines and tumors overexpress *PTCH1*, a readout of Hedgehog signaling, with *PTCH1* mRNA expression increasing from normal ICC to GIST. We also detected inactivating *PTCH1* mutations in nearly 2% of genomically sequenced tumors, and nearly 10% of all GIST studied had one or more potentially deleterious variants in *PTCH1* and other Hedgehog pathway genes. While most appeared to represent passenger mutations, three tumors lacked known driver mutations in *KIT*, *PDGFRA*, *BRAF*, *KRAS*, *HRAS*, *NF1*, and *SDH* subunits but had potentially deleterious variants in *GLI1* and *SUFU*. However, the true significance of these mutations is unclear as their oncogenic capacity was only assessed bioinformatically, but not experimentally.

The Hedgehog pathway is one of several developmental signaling pathways known to be important in tumorigenesis. Several of the Hedgehog-related genes overexpressed in GIST are involved in regulating cell proliferation (*CD3E*, *HIPK1/2*, and *PTCH1*), cell differentiation (*CDON* and *FOXF1*) and apoptosis (*CD3E* and *HIPK1/2*). Other genes include regulators of primary cilia formation (*C2CD3*, *KIF3A*, *IFT172*, *IFT52*, and *IFT88*), and negative regulators of the Hedgehog pathway (*HHIP*, *RAB23*, and *TULP3*). Developmental signaling pathways tend to intersect. Indeed, several of the altered transcripts in GIST are also related to the Wnt, Notch, BMP and FGF pathways. The Wnt and Notch pathways are important in GIST biology [40, 41], and collagen triple helix repeat containing 1 (*CTHRC1*), an activator of Wnt/planar cell polarity-Rho signaling, was shown to induce GIST migration and invasion, as well as correlated with recurrence risk score, disease-free survival and overall survival in 412 GIST patients [40]. *HES1*, a Notch and Hedgehog pathway target gene known to suppress gene transcription, was found to induce growth arrest and decrease *KIT* expression, reducing GIST viability [41]. We now demonstrate that the Hedgehog pathway in ICC and GIST also includes GLI3R-mediated transcriptional repression of *KIT*, which appears to represent an internal “brake” mechanism limiting the effects of GLI1/2-induced *KIT* expression. Similarly to blocking the activating GLI1/2 isoforms, increasing GLI3R inhibited *KIT* expression, while blocking *GLI3* mRNA increased *KIT* expression. These findings, together with our results showing that GLI1/2 inhibition by ATO increased *KIT* promoter occupancy by GLI3 indicates that the Hedgehog pathway likely regulates *KIT* transcription by a “push-pull” mechanism. These findings are consistent with our gene expression data and an earlier report in mouse ureteral ICC-like cells [20]. Molecular progression of GIST involves sequential accumulation of chromosomal losses and gains [42, 43], including frequent 7p13 amplification associated with *GLI3* overexpression in nearly 20% of tumors [19]. Dominance of GLI3-mediated transcriptional repression may contribute to reduced or lost *KIT* expression seen in a subset of TKI-treated GIST and in ICC/GIST precursors [3–5].

We also found GIST to highly express four genes (*KIF3A*, *IFT52*, *IFT88*, and *IFT172*), which are associated with PC function in several cancers. Conditional deletion of *KIF3A* or *IFT88* in basal cell carcinoma leads to ciliary elimination and strong inhibition of tumorigenesis induced by mutant SMO [44]. In contrast, ablation of PC enhances brain tumor growth induced by activated GLI2 [45]. These paradoxical effects are consistent with a dual opposing function of PC in regulating Hedgehog signal transduction [44]. We here show that GIST cells lack expression of Hedgehog ligands, but overexpress *GLI2* in the presence of PC, which activates the Hedgehog pathway [22]. In this context, the PC produces the repressor form of GLI (i.e., GLI3R) in order to counterbalance GLI2 activation. Under the control of the ICC and GIST marker, anoctamin 1 (ANO1) [27], the function of the PC is mediated by the IFT machinery, which supports Hedgehog and PDGFRA signal transduction along the axoneme [22]. For IFT trafficking to occur, the microtubule plus-end directed motor kinesin-II (including KIF3A) and the IFT-B protein complex (including IFT52, IFT88, and IFT172) are necessary. We now show that these PC genes are expressed in GIST, consistent with previous reports that primary, recurrent, and metastatic GIST have PC [25, 26]. Thus, ICC and GIST have PC, which are important for Hedgehog and PDGFRA signaling; and GIST overexpress critical PC machinery involved in normal and aberrant PC signaling.

In conclusion, our findings provide a new and expanded understanding for the role of the Hedgehog pathway in the development and progression of GIST. We now provide the first evidence that Hedgehog developmental signaling is present in the ICC lineage and is dysregulated in GIST, irrespective of *KIT*/*PDGFRA* mutations or tumor location, adding Hedgehog to the growing list of developmental signaling pathways important in GIST. Further studies will define the utility of Hedgehog-related pharmacological agents in the treatment of imatinib-resistant GIST.

## MATERIALS AND METHODS

### Human GIST Samples

After obtaining informed consent, tumor samples were collected from 3 GIST patients undergoing resections at the University of California, San Diego (UCSD). All procedures were approved by the UCSD Institutional Review Board (IRB) (#090401). Pathological diagnosis was made by an experienced pathologist based on light microscopic analysis of formalin-fixed, paraffin-embedded (FFPE) tissue sections and sections labeled with antibodies against KIT and ANO1. *KIT* and *PDGFRA* activating mutations were analyzed by PCR and sequencing (ARUP Laboratories, Salt Lake City, UT). Excess tumor tissue was used for research purposes.

### Tissue Source of Human ICC and Isolation by Fluorescence-Activated Cell Sorting (FACS)

De-identified human gastric tissues that were used for the preparation of primary ICC were obtained as surgical excess tissue from 14 patients undergoing bariatric operations (see Supplementary Materials and Methods) [46]. All procedures including waiver of the consent were approved by the Mayo Clinic IRB (protocol #07-003371). The tissues were dissected, dissociated and labeled using previously published protocols [47] modified for human samples.

### RNA Preparation from ICC Isolates for Microarray Analysis

Total RNA was prepared from FACS-purified human ICC using the Total RNA Purification Kit (Norgen Biotek Corp, Thorold, ON, Canada) and purified using the MinElute Reaction Cleanup Kit (Qiagen, Hilden, Germany). RNA quality was assessed with the Agilent RNA 6000 Pico Kit (Agilent, Waldbronn, Germany). The Ovation Pico WTA system (NuGEN, San Carlos, CA) was used to prepare cDNA by single primer isothermal amplification (SPIA^®^) for hybridization to the Affymetrix GeneChip Human Genome U133 Plus 2.0 array (Affymetrix, Santa Clara, CA).

### Sources of Human GIST and ICC-related Microarray Data

The human GIST microarray data from 69 patients discussed in the current study were retrieved from the National Center for Biotechnology Information Gene Expression Omnibus (NCBI GEO;http://www.ncbi.nlm.nih.gov/geo/) and are accessible through GEO Series accession numbers GSE17743 [32] (n=29 gastric GIST), GSE8167 [33] (n=23 gastric GIST), and GSE20708 [19] (n=17 gastric GIST) (see Supplementary Table S2 for details). Microarray data from FACS-sorted human ICC (n=6), HP^+^ cells (n=3), and *NOT* ICC (n=1), as well as unfractionated gastric *tunica muscularis* tissues (n=4) generated in this study are accessible through GEO SuperSeries accession number GSE77839. Microarray data previously generated from mouse small intestinal myenteric ICC (ICC-MY) and deep muscular plexus ICC (ICC-DMP), as well as their source tissues are accessible through GSE7809; gene expression data from 2XSCS2F10 ICC-SC and their source tissue are accessible through GSE60854.

### Analysis of Human Gastric GIST and ICC Microarray Data

Gene expression was compared between human gastric ICC, human gastric GIST, mouse gastric 2xSCS2F10 ICC-SC, mouse intestinal ICC-MY/ICC-DMP and their respective unfractionated source tissues, as well as between human gastric ICC and gastric *KIT*-mutant GIST from GSE17743 (n=15). Probe-level data were pre-processed by robust multiple-array analysis (RMA) and analyzed for differential gene expression by the empirical Bayes approach with Benjamini-Hochberg adjustment using software packages in Bioconductor (https://www.bioconductor.org/[30]). Differential expression with log_2_ fold change <−1 or >1 and *Q*<0.05 was considered significant. Unique gene lists were created by identifying the probe sets with the lowest Benjamini-Hochberg false discovery rate *Q*-values. Process networks in gene sets significantly overexpressed in human and mouse ICC and mouse ICC-SC were identified by MetaCore™ analysis (GeneGo, Thomson Reuters Corp., New York). Gene expression data for human GIST (GSE17743, GSE8167, and GSE20708), purified human ICC, *NOT* ICC, HP^+^ cells, as well as human gastric *tunica muscularis* tissues were also quantified using the MAS5 algorithm in the Bioconductor package *affy*. Significance of expression was determined by Wilcoxon signed-rank test, and heat maps were assembled using probe set expression values with the lowest *P*-value. Unsupervised hierarchical clustering was performed using *gplots*.

### Cell Culture

We obtained the GIST-T1 line containing a *KIT* exon 11 (V560-Y579Δ5: imatinib-sensitive) mutation [48] from Dr. T. Taguchi (Kochi Medical School, Japan), the GIST882 line containing a *KIT* exon 13 (K642E: imatinib-sensitive) mutation [49] from Dr. S. Singer (Memorial Sloan-Kettering Cancer Center, New York), and the GIST48IM lines containing *KIT* exon 11 (homozygous V560D: imatinib-sensitive) and *KIT* exon 17 (heterozygous D820A: imatinib-resistant) mutations from Dr. J. Fletcher (Dana-Farber Cancer Center, Boston, MA) [50]. All cell lines were cultured as previously reported. HEK293T cells were grown in DMEM with 10% FBS, 1% penicillin/streptomycin (Mediatech), and 2 mM glutamine (Mediatech).

### Statistical Analysis

Statistical analyses were performed using GraphPad Prism 4 (GraphPad Software, La Jolla, CA) or SigmaPlot 10 (with SigmaStat plug-in; Systat Software, Inc., San Jose, CA). Results are expressed as the mean ± SEM or SD as appropriate. Comparisons between two groups were performed using the Student's *t*-test (Stata 9.0, StataCorp, College Station, TX). Statistical significance was accepted at the 5% level.

### Other Materials and Methods

Standard methods (i.e., real-time reverse-transcription PCR, transient transfection by electroporation, Western blotting, chromatin immunoprecipitation, and cell viability assays) and additional details are described in the Supplementary Materials and Methods.

## SUPPLEMENTARY MATERIAL






