# Supplementary material for: Hedgehog pathway dysregulation contributes to the pathogenesis of human gastrointestinal stromal tumors via GLI-mediated activation of KIT expression
Source: Oncotarget. 2016 Oct 25;7(48):78226–41. doi: 10.18632/oncotarget.12909 (PMC5346634; doi:10.18632/oncotarget.12909)
Supplement: Supplementary file 1 [file oncotarget-07-78226-s001.pdf]

Hedgehog pathway dysregulation contributes to the pathogenesis of human gastrointestinal stromal tumors *via* GLI-mediated activation of *KIT* expression

Supplementary Material

Animal Studies

Transgenic mice carrying the activating *KIT* mutations $Kit^{V558\Delta}$ [1] and Kit^{K641E} [2] were obtained from breeders kindly provided by Dr. P. Besmer (Memorial Sloan-Kettering Cancer Center, New York) and Dr. B. Rubin (Cleveland Clinic, Cleveland, OH), respectively. Mice were housed in a barrier facility on a 12-hour light-dark cycle, and autoclaved laboratory rodent chow and water were available *ad libitum*. The UCSD Institutional Animal Care and Use Committee approved all experimental procedures. Animal studies were conducted in accordance with USPHS Policy on Humane Care and Use of Laboratory Animals.

Comprehensive Genomic Profiling and Data Analysis

De-identified tumor samples (n=191) from 186 unique patients were selected from the Foundation Medicine (FM), Inc. (Cambridge, MA) database consisting of patients from across the world who underwent genomic profiling during the course of their clinical care. De-identified tumor samples were selected from the database of patients who underwent FoundationOne® analyses from October 2012 to May 2015. We retrospectively analyzed this prospectively collected data under a UCSD IRB approved protocol (#141555X). The genomic profiling as previously reported was performed on 191 tumor samples from 186 patients [3].

The GIST DNA was extracted from formalin-fixed paraffin embedded (FFPE) tumor specimens taken from either primary or secondary tumor biopsies and submitted to a Clinical Laboratory Improvement Act certified , New York Stage accredited, and College of American Pathologists-

accredited laboratory, Foundation Medicine, Inc. (FM), by healthcare providers from various medical institutions or community hospitals. Comprehensive genomic profiling (CGP) was performed on hybridization-captured, adaptor ligation-based libraries using DNA extracted from four FFPE sections cut at 10 μm from the tumors. All samples sent for DNA extraction contained a minimum of 20% nuclei derived from tumor cells. The FoundationOne® assay is a next-generation genomic assay that utilizes the Illumina HiSeq 2500 instrument (Illumina Inc., San Diego, CA) to sequence against hybridization-captured, adaptor ligation-based libraries for coding regions of 315 cancer-related genes plus introns from 28 genes frequently implicated in cancer transformation. This assay simultaneously analyzes the extracted DNA for base substitutions, short insertions and deletions, amplifications and homozygous deletions and gene fusions and rearrangements as previously described [3]. The average sequencing depth for the 191 GIST was 585-fold and for the 19 GIST with Hedgehog-related alterations in *PTCH1*, *PTCH2*, *SMO*, *GLI1*, and *SUFU* was 552-fold.

The genomic alterations were identified using Foundation Medicine's workflow [3]. To maximize sensitivity in heterogeneous GIST specimens, the test was validated to detect base substitutions, as well as short insertions and deletions at ≥10% mutant allele frequency with ≥99% sensitivity. The genomic alterations were further categorized (i.e., known somatic, likely somatic, or variant of unknown significance, VUS) using public and custom annotation tools. Furthermore, point mutations and small indels were annotated for their functional effect on the protein (missense, nonsense, frameshift, etc.). In order to expand our understanding of the potential deleterious effect of missense VUS's, we mapped them to the dbNSFP database, which pre-computed multiple scores for all possible base substitution in coding regions.[4, 5] VUS's that were predicted deleterious by at least 2 out of 4 prediction tools (SIFT, PolyPhen, MutationTaster, and/or MutationAssessor) were included in further analyses. Any missense VUS that did not meet this criterion was excluded. Copy number alterations, gene fusions, and

gene rearrangements were considered known/likely genomic alterations based upon their detection with CGP

As the FM sequencing process does not include a matched germline, it is possible for an inherited variant to be erroneously called somatic. Inherited variants are detected in the assay, but not classified as such in the report. The Exome Aggregation Consortium (ExAC) Browser (exac.broadinstitute.org) provides a reliable registry of all known human germline variations down to a minor allele frequency of 10^{-5} [6]. We were able to match 495/1607 (30.8%) of the short variants to the same residue, as well as with the same amino-acid substitution in the ExAC database (version 0.3) using the protein annotation. Mutations present at a minor allele frequency greater than 1% indicating potential germline variants were excluded from analysis.

Isolation of Human Interstitial Cells of Cajal (ICC) by Fluorescence-activated Cell Sorting (FACS)

Human gastric tissue representing 1-84 cm^2 area the greater curvature was pinned flat in a dissection dish coated with Sylgard 184 silicone elastomer (Dow Corning, Midland, MI) and the mucosa was removed by peeling. The tissues were cut into small pieces and incubated with Human Fc Receptor Binding Inhibitor (20 µL/mL; eBioscience, Inc., San Diego, CA) and allophycocyanin (APC)-conjugated mouse anti-human Kit antibody [1 µg/mL in Ham's F12 Nutrient Mixture (Thermo Fisher Scientific, Waltham, MA), containing 10% FBS; to label ICC in situ; BioLegend, Inc., San Diego, CA; see detailed antibody information in **Supplementary Table S3**) for up to 9 h at 4 °C on a rocker. The tissue was then transferred into collagenase-containing enzyme solution [7] and incubated at room temperature for up to 9 hours with shaking. After sedimentation by short centrifugation, the collagenase solution was replaced with Ham's F12 Nutrient Mixture containing 10% FBS and the tissue was dissociated by gentle

trituration using a series of 3-4 fire-polished glass pipettes of decreasing tip diameter [7]. The cell suspension was then washed in PBS containing 10% FBS and filtered through a 500-µm and then a 100-µm nylon mesh filter (Corning Corp., Corning, NY) to obtain single-cell suspensions. Red blood cells were lysed with ACK (Ammonium-Chloride-Potassium) Lysing Buffer (5 mL, room temperature for 5 min; Thermo Fisher Scientific, Waltham, MA). After adding 10 mL PBS, the cell suspension was filtered through a 40-µm nylon mesh filter (Corning Corp., Corning, NY), washed with PBS and counted.

Dead cells were labeled with Fixable Viability Dye APC-eFluor 780 (1 µL/mL, 4°C for 30 min; eBioscience, Inc., San Diego, CA). After washing with Ham's F12 Nutrient Mixture containing 10% FBS, cells were resuspended in 100 µL of the same medium and incubated on ice with Human Fc Receptor Binding Inhibitor (20 µL, 30 min). Immunolabeling was performed on ice for 45 min with the antibodies listed in **Supplementary Table S3**: Hematopoietic (HP$^+$) cells were identified with a cocktail of antibodies conjugated with phycoerythrin-cyanine 7 (PE-Cy7) including anti-CD45 (PTPRC, protein tyrosine phosphatase, receptor type, C; to label all leukocytes; BD Pharmingen, Becton, Dickinson and Company, Franklin Lakes, NJ), anti-FCER1A (high affinity I receptor for Fc fragment of IgE, alpha polypeptide; to identify mast cells; BioLegend, Inc., San Diego, CA), and anti-CD11B (ITGAM, integrin, alpha M; to label monocytes/macrophages, neutrophils and eosinophils; BD Pharmingen, Becton, Dickinson and Company, Franklin Lakes, NJ). Detection of ICC (HP$^-$KIT$^+$CD34$^-$ cells) and *NOT* ICC (HP$^-$KIT$^-$CD34$^-$ cells) was facilitated by labeling with APC-anti-KIT (BioLegend, Inc., San Diego, CA) and PE-anti-CD34 (BD Pharmingen, Becton, Dickinson and Company, Franklin Lakes, NJ) antibodies. Additional aliquots of the same samples were labeled in an identical manner but with isotype controls for the anti-KIT or the anti-CD34 or the anti-hematopoietic marker antibodies. Our experimental design was also extensively verified by using single-stained and fluorescence-minus-one controls. The cells were then washed with PBS containing 5% FBS twice and

resuspended in 350 µl of PBS containing 5% FBS. Samples were analyzed using a Becton Dickinson (BD Bioscience, Franklin Lakes, NJ) LSR II flow cytometer (see configuration in **Supplementary Table S4**) and FlowJo software (Treestar, Ashland, OR) (see **Supplementary Figure S4** for gating scheme and representative projections). ICC, *NOT* ICC, and HP^+ cells (including KIT^+ mast cells and other hematopoietic cells) were isolated by FACS using a Becton Dickinson FACS Aria II Cell Sorter configured to match the LSR II cytometer (excitation wavelengths of the lasers utilized: 488 nm and 635 nm). Sorting fidelity was analyzed by microarray and real-time reverse-transcription PCR (qRT-PCR; **Supplementary Figure S5**). Harvested cell counts are shown in **Supplementary Table S5**. Freshly dissociated, unfractionated *tunica muscularis* tissues were processed as additional reference.

Two-step Real-time qRT-PCR

Human and mouse tumor tissues were homogenized in TRIzol reagent (Life Technologies, Carlsbad, CA) with a tissue homogenizer for 1 min at maximum speed. Total RNA from these homogenates or cell pellets was extracted using TRIzol reagent (Life Technologies) and reverse transcribed into cDNA with iScript cDNA synthesis kit (Bio-Rad Laboratories, Hercules, CA) according to the manufacturers' instructions. Quantitative real-time PCR was conducted with SsoFast EvaGreen Supermix (Bio-Rad Laboratories) on a CFX96 real-time system (Bio-Rad Laboratories) using primers specific for genes of interest and the β-actin (*ACTB*) housekeeping gene. Primers were designed using GenBank sequences or as previously described [8, 9]. Primer sequences for human and mouse genes assayed are listed in **Supplementary Tables S6** and **S7**, respectively. Samples were run in triplicate with the following PCR parameters: denaturing at 95°C for 30-s followed by 40 cycles of 5-s denaturation at 95°C and 5-s annealing–extension at the optimal annealing temperature for each primer. The CFX96 system automatically generated threshold cycle (Ct) values after each run. Target gene levels in cells

are presented as a ratio to levels of *ACTB* according to the ΔCt method, or as a ratio to levels detected in the corresponding control cells according to the ΔΔCt method [10]. These fold changes were determined using point and interval estimates. Final PCR products were analyzed by gel electrophoresis separated on a 2.0% agarose gel run in 1X Tris-acetate-EDTA buffer.

Chromatin immunoprecipitation—real-time PCR (ChIP-qPCR)

GIST-T1 and GIST-882 cells were cultured with media containing 4 μM arsenic trioxide or vehicle control for 48 h. The cells were treated with 1% formaldehyde (Thermo Scientific, Waltham, MA) for 10 min at room temperature to cross-link DNA–protein complexes. The reaction was quenched with 125 mM glycine for 5 min at room temperature. Cells were then lysed for 10 min at 4 °C with a lysis buffer containing 10 mM Tris-HCl, pH 7.5, 10 mM NaCl, and 0.5% NP-40. Chromatin was first fragmented at 37 °C for 20 min with micrococcal nuclease at 2000 gel units/mL (New England Biolabs, Ipswich, MA) in a digestion buffer containing 20 mM Tris-HCl, pH 7.5, 15 mM NaCl, 60 mM KCl, and 1 mM $CaCl_2$. The digestion was stopped with a reaction buffer containing 100 mM Tris-HCl, pH 8.1, 20 mM EDTA, 200 mM NaCl, 2% Triton X-100, and 0.2% sodium deoxycholate. The chromatin was further fragmented for 15 cycles of 30 s on and 30 s off using a Bioruptor sonicator (Diagenode, Denville, NJ). Chromatin preparations were incubated with anti-GLI3 antibody (Santa Cruz Biotechnology, Inc., Dallas, TX) overnight at 4 °C and precipitated using Protein-G agarose beads (Roche Diagnostics, Indianapolis, IN). After elution of DNA-protein complexes, crosslinks were reversed by overnight 65-°C heat treatment, and proteins were degraded with proteinase K (Thermo Scientific) for 2 h at 37°C. DNA was purified using Qiagen PCR Purification Kit (Valencia, CA). Enrichment of target DNA relative to 1% of input DNA was analyzed by qPCR using primers to amplify regions surrounding the transcription start site (TSS) of *KIT* promoter. Primer sequences are the following: TSS1 for the region encompassing the TSS (F: GGATCCCATCGCAGCTACCG; R:

CCTGTCTGGACG CGAAGCAG); and TSS2 for the region immediately upstream of the TSS (F: GCCACGC CGACCAATAGGAA; R: AAAGGCTGAGCCCGGTTTCG) and TSS3 for the region downstream of the TSS (F: CGAATCCGGGGTTCTTCGGG; R: AAAGCCACCCCAAACTCGCA).

Transient Transfection by Electroporation

Using the Amaxa® Cell Line Nucleofector® Kit V (Lonza, Cologne, Germany), GIST882 cells were electroporated (n=3) using 2 million cells per condition per assay according to manufacturer's instructions. The following siRNA oligos selected from siGENOME SMARTpool (GE Dharmacon, Lafayette, CO, USA) were electroporated for knockdown experiments (2 µM each): *GLI1* (M-003896-00-0005), *GLI2* (M-006468-02-0005) and *GLI3* (M-011043-01-0005). Scrambled oligos (siGENOME non-targeting siRNA pool #2) were used as a negative control. *In vitro* overexpression was generated by electroporating with either 4 µg of $GLI3^R$ plasmid DNA or a pGIPZ lentiviral empty vector (Open Biosystems, Huntsville, AL) [11]. Cells were incubated for 48 hours before assessing the outcome of transfections (n=3).

Western Blotting

Cells were lysed with RIPA buffer (Cell Signaling Technology, Inc., Danvers, MA) containing Halt Protease Inhibitor Cocktail (Pierce, Thermo Fisher). Cell lysate concentration was determined using a DC protein assay (Bio-Rad). 50 µg lysates were separated on a 4-12% NuPAGE BIS-Tris gel (Life Technologies), and transferred using I-Blot dry blotting system (Life Technologies). The PVDF membrane was blocked with 5% (w/v) BSA in TBST buffer (i.e. Tris-buffered saline and 0.1% (v/v) Tween-20) for 1 h at room temperature. Then the membrane was incubated overnight at 4°C with antibodies against GLI3 protein (1:1000, Santa Cruz Biotechnology) or β-actin protein (1:1000, Cell Signaling). After washing 4 times with TBST

buffer at room temperature for 5 minutes each, the membrane was incubated at room temperature for 1 h with a secondary antibody HRP-conjugated goat anti-rabbit IgG (1:10,000 in TBST buffer with 5% (w/v) nonfat milk, Thermo Scientific) followed by additional 4 TBST washes. Protein signals were detected using the Pierce ECL Western Blotting Substrate (Thermo Scientific).

Pharmacological GLI1/2 Inhibition and Cell Viability Assay

GIST-T1, GIST882 and GIST48IM cells were treated for 48h with 10 μM GANT61 (Cayman Chemical, Ann Arbor, MI), 4 μM arsenic trioxide (ATO, Sigma-Aldrich), 10 μM imatinib (Chemietek, Indianapolis, IN) or 10 μM sorafenib (Chemietek). Control experiments have indicated that high levels (>0.1%) of dimethyl sulfoxide (DMSO, Sigma-Aldrich, St. Louis, MO) induce cell death in GIST cells (data not shown). Hence, cells treated with 0.1% DMSO only were used as controls for testing compounds that were dissolved in DMSO. *KIT* expression was assessed by qRT-PCR as described above. In the dose-response studies, GIST-T1, GIST882 and GIST48IM cells were seeded in 96-well plates and treated in triplicate with 10 μM-169 pM ATO or imatinib for 72, 120 h and 120 h, respectively. Methyl-tetrazolium (MTT) assay was employed to determine cell viability by adding MTT reagent, 3-(4, 5-dimethylthiazol-2-yl)-2,5-diphenyltetrazolium bromide (Sigma-Aldrich), and then incubating for 4 h at 37°C. DMSO was used to dissolve the purple formazan product. The 96-well plate was read to determine absorbance at 570 nm. The background absorbance was determined from wells with culturing media only; and wells containing control cells were set as 100% viability.

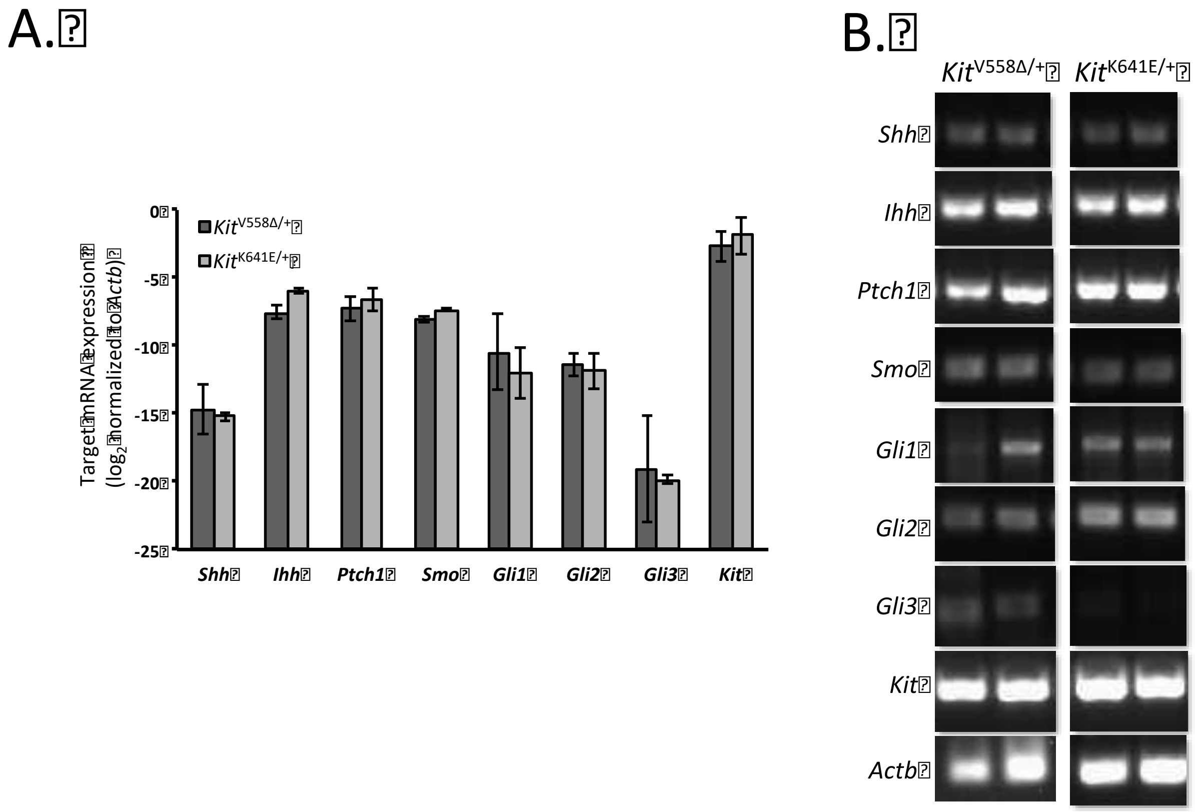

Supplementary Figure S1. Expression of Hedgehog signaling components in tumors harvested from two different GIST-bearing transgenic mouse strains. **A**, We studied *Kit*$^{V558\Delta/+}$ [1] and *Kit*$^{K641E/+}$ [2] knock-in transgenic mice that develop cecal GIST. Total RNA was extracted from GIST (n=3 mice/model). qRT-PCR analysis was performed for Hedgehog signaling components. mRNA expression was quantified by the ΔCt method using *ACTB* as reference. **B**, Agarose gel electrophoresis of two-step qRT-PCR products from two different mice shows expression of Hedgehog signaling components.

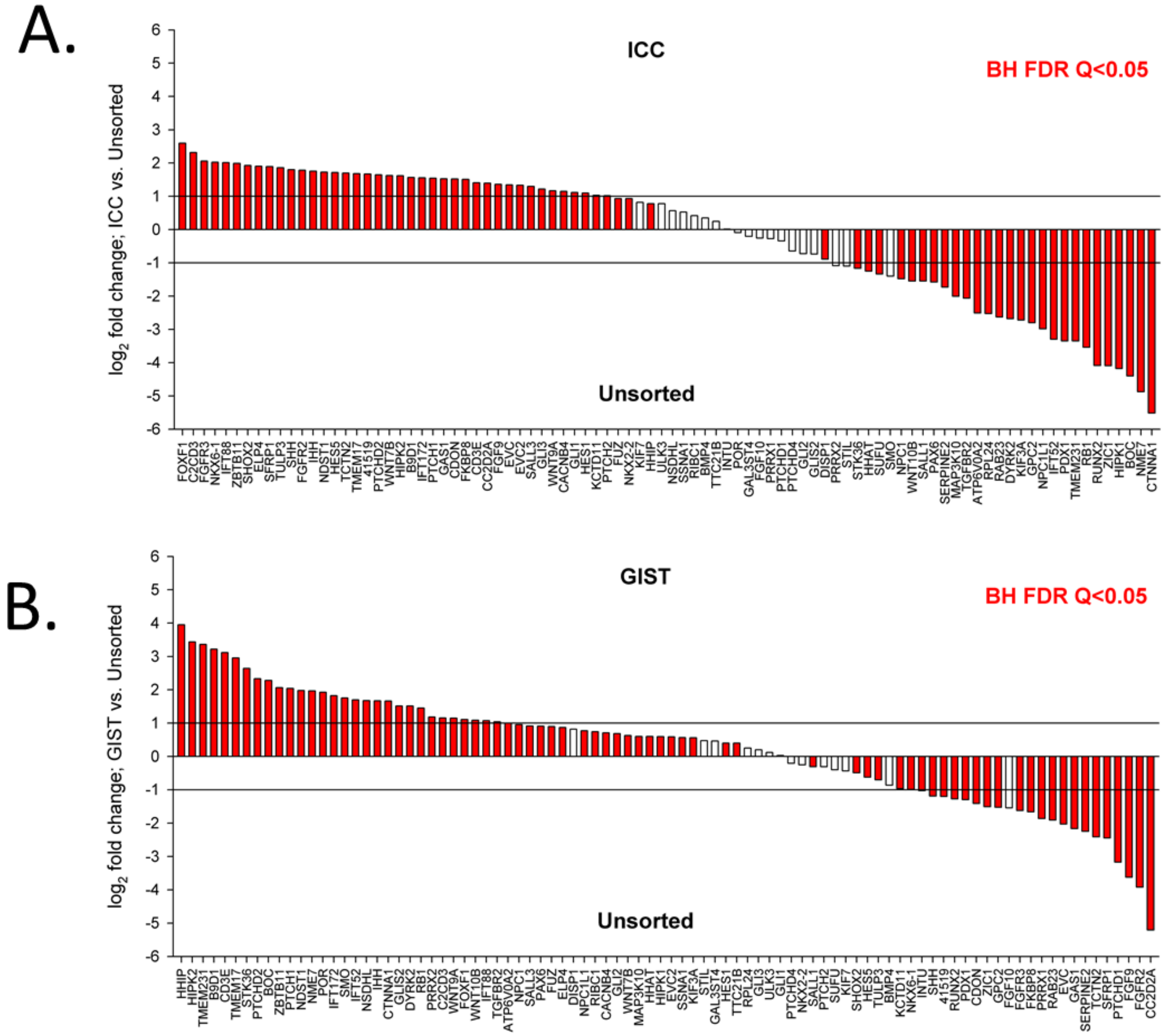

Supplementary Figure S2. Differential expression of Hedgehog signaling-related genes detected by microarray analysis in human gastric *KIT*-mutant GIST, purified human ICC and their source tissue (unfractionated gastric *tunica muscularis*). The list of Hedgehog-signaling-related genes was assembled by searching the Affymetrix Human Genome U133 Plus 2.0 microarray annotation file (na33) for gene ontology terms containing “smoothened” (biological process) and “Hedgehog” (molecular function). Differential gene expression was determined as described in the **Materials and Methods**. Unique gene lists were created by identifying the probe sets with the lowest Benjamini-Hochberg false discovery rate (BH FDR) *Q* values. **A**. ICC (n=6) compared to source tissues (n=4). **B**. *KIT*-mutant gastric GIST from the National Center for Biotechnology Information Gene Expression Omnibus Series GSE17743 [12] (n=15) compared to tissue samples (n=4). Horizontal lines indicate threshold for significant $\log_2$ fold changes; red fill indicates Q<0.05 from BH FDR. Differential expression with $\log_2$ fold change <-1 or >1 and Q<0.05 was considered significant.

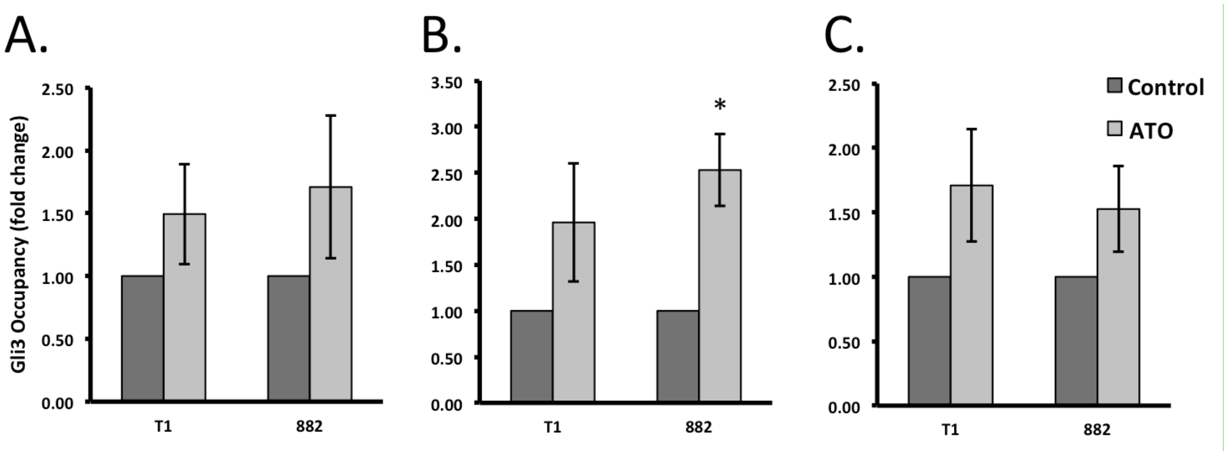

Supplementary Figure S3. GLI3 binding to the *KIT* promoter detected by chromatin immunoprecipitation is increased by treatment with the GLI1/2 inhibitor arsenic trioxide (ATO). GIST-T1 and GIST882 cells were treated with 4 μM ATO or vehicle control for 48 hours. Chromatin fragments immunoprecipitated by anti-GLI3 antibodies were amplified by real-time PCR to analyze GLI3 binding to the *KIT* promoter region. **A.** GLI3 binding to TSS1, the region encompassing the transcription start site (TSS). **B.** GLI3 binding to TSS2, the region immediately upstream of the TSS. **C.** GLI3 binding to TSS3, the region immediately downstream of the TSS. GLI3 binding to the TSS2 region of the *KIT* promoter was increased 2.5-fold with ATO treatment in GIST882 cells (**S3B,***P<0.02). In other regions and in GIST-T1 cells, GLI3 binding showed trends towards increased promoter binding with ATO treatment.

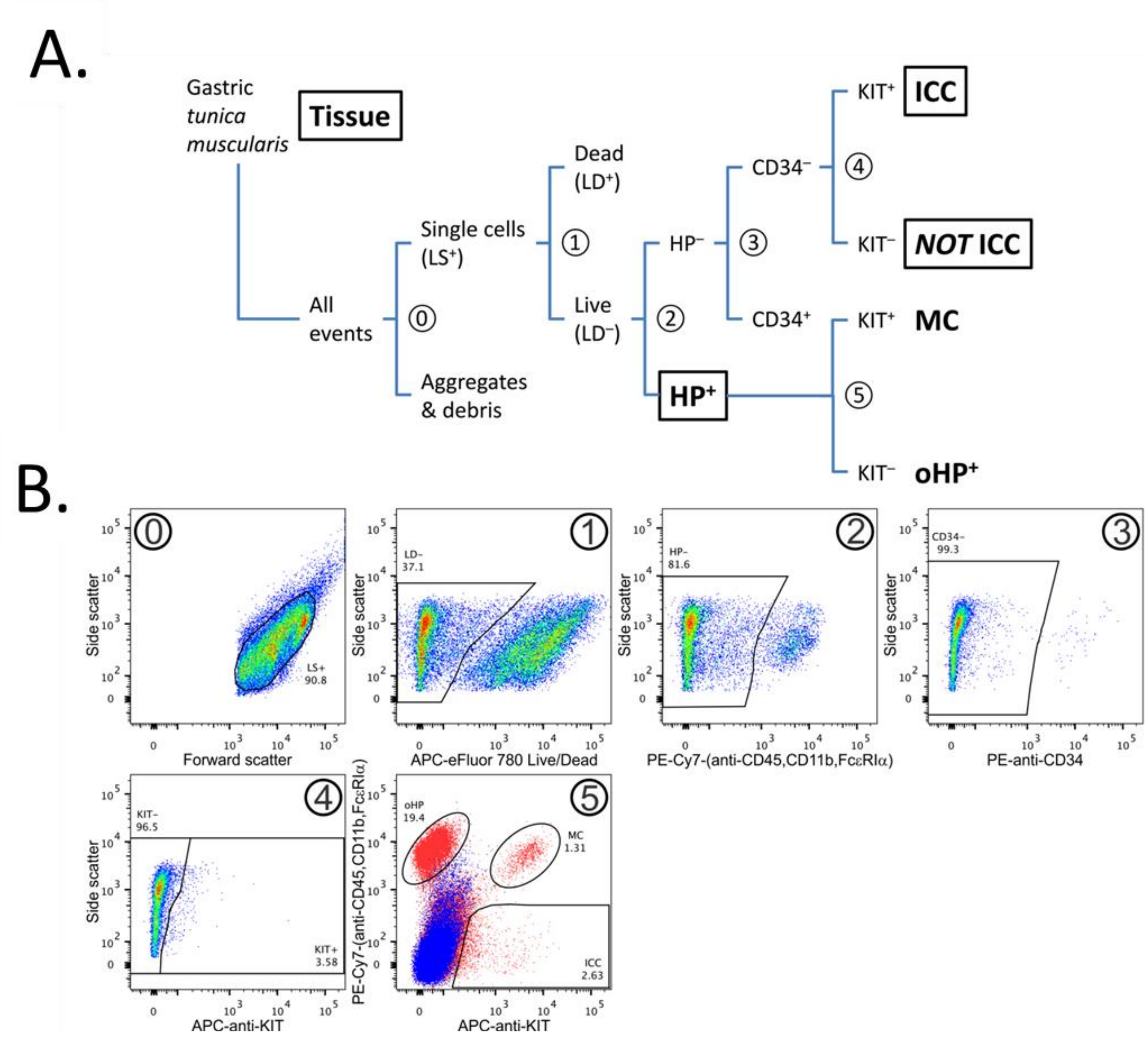

Supplementary Figure S4. Purification of ICC, *NOT* ICC and hematopoietic cells from the human gastric *tunica muscularis* by FACS. A, Gating sequence. Dead cells were marked and excluded from further analysis with the Fixable Viability Dye allophycocyanin (APC)-eFluor 780 (FVD^+). Hematopoietic (HP^+) cells were identified with a cocktail of antibodies conjugated with phycoerythrin-cyanine 7 (PE-Cy7) including anti-CD45 (PTPRC, protein tyrosine phosphatase, receptor type, C; to label all leukocytes), anti-FCER1A (high affinity I receptor for Fc fragment of IgE, alpha polypeptide; to identify mast cells, FcεR1α), and anti-CD11B (ITGAM, integrin, alpha M; to label monocytes/macrophages, neutrophils and eosinophils). Detection of ICC (defined as $HP^-KIT^+CD34^-$ cells) and *NOT* ICC (defined as $HP^-KIT^-CD34^-$ cells) was facilitated by labeling with APC-anti-KIT and PE-anti-CD34 antibodies. MC, mast cells; oHP^+, other hematopoietic cells (excluding mast cells). Boxed labels indicate sorted (ICC, *NOT* ICC, HP^+) and unsorted cells (Tissue) used in the microarray studies. **B**, Representative projections illustrating key cell populations identified by circled numbers in A. Population boundaries were drawn along lines representing identical event distribution probabilities in contour plots. Numbers in the two-dimensional projections indicate the proportion of cells within the specified clusters as percentages of the total cell count in the projection. ⓪: Detection of cells with light

scatter properties characteristic of single live cells (LS$^+$). ①: Exclusion of dead cells identified by their uptake of FVD APC-eFluor 780 (FVD$^+$). ②: Separation of cells of hematopoietic origin (CD45$^+$FcεR1α$^+$CD11b$^+$ leukocytes; HP$^+$) from other components of the gastric muscles (HP$^–$). ③: Detection of CD34$^–$ cells in the HP$^–$population. ④: Separation of KIT$^+$ ICC from KIT$^–$ *NOT* ICC in the HP$^–$C34$^–$population. ⑤: Separation of KIT$^+$ MC from the oHP$^+$ population and from HP$^–$KIT$^+$ cells containing ICC. Dot plot overlay of a sample stained with all antibodies indicated above and a corresponding sample that was labeled with a full set of isotype control antibodies. Note high number and strong KIT immunoreactivity of MC and their separation from ICC.

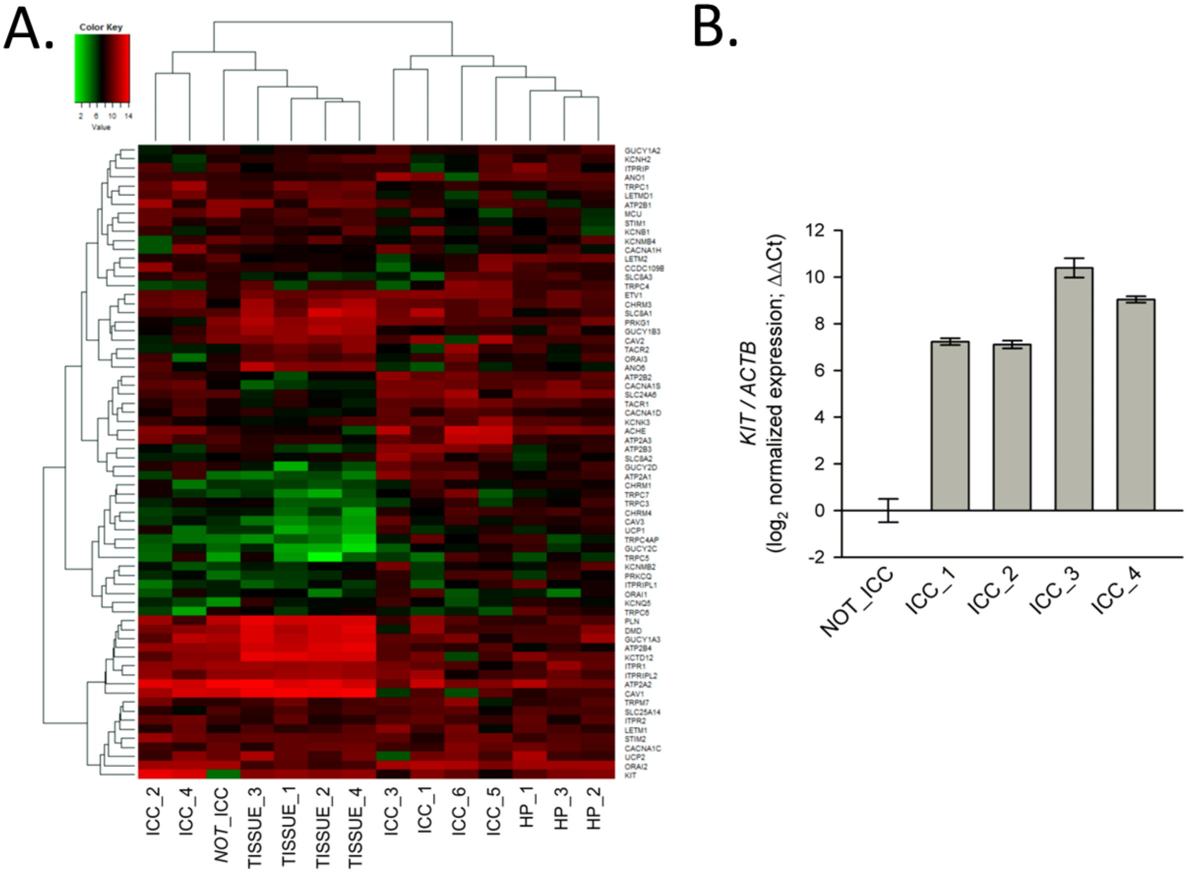

Supplementary Figure S5. Validation of cell sorting by microarray and real-time quantitative reverse transcription-PCR (qRT-PCR) analysis. A, Analysis of FACS-sorted ICC, *NOT* ICC and hematopoietic cells (HP), as well as unfractionated gastric *tunica muscularis* tissues (Tissue) by Affymetrix, Inc. (Santa Clara, CA) Human Genome U133 Plus 2.0 microarrays. ICC-related genes were identified based on the literature [13-26]. Expression values were determined using the MAS5 algorithm in the Bioconductor (https://www.bioconductor.org/) *affy* package [27]. Significance of expression was determined by Wilcoxon signed-rank test. Heat maps were assembled using probe set expression values with the lowest *P* value and analyzed by hierarchical clustering. All arrays passed hybridization quality control including background, scale factor, and 3'/5' ratios. ICC microarrays appeared in two clusters possibly reflecting regional heterogeneity of ICC [13, 14, 28]. Sample-to-sample variability may have also reflected amplification bias due to low input. Therefore, we further analyzed expression of key ICC genes (*ETV1*, *KIT*, *ANO1*, *PRKCQ*, *and TRPM7*) relative to the average expression in the Tissue samples. In each sample at least 2 of these genes were overexpressed >1.5-fold. Most consistent increased expression was detected in *ANO1* (5/6), *PRKCQ* (4/6) and *ETV1* (4/6; >1.36). Relative *KIT* expression was lower probably reflecting the presence of KIT^{high} mast cells in the Tissue samples (see **Supplementary Figure 4B**, panel ⑤). Therefore, we compared *KIT* expression by qRT-PCR in ICC samples where sufficient cDNA was available using *NOT* ICC as reference (**B**). These results indicated strong KIT expression relative to the samples depleted of ICC and mast cells.

References:

1. Rossi F, Ehlers I, Agosti V, Socci ND, Viale A, Sommer G, Yozgat Y, Manova K, Antonescu CR, Besmer P. Oncogenic Kit signaling and therapeutic intervention in a mouse model of gastrointestinal stromal tumor. Proc Natl Acad Sci U S A. 2006; 103: 12843-8. doi: 10.1073/pnas.0511076103.
2. Rubin BP, Antonescu CR, Scott-Browne JP, Comstock ML, Gu Y, Tanas MR, Ware CB, Woodell J. A knock-in mouse model of gastrointestinal stromal tumor harboring kit K641E. Cancer Res. 2005; 65: 6631-9. doi: 10.1158/0008-5472.CAN-05-0891.
3. Frampton GM, Fichtenholtz A, Otto GA, Wang K, Downing SR, He J, Schnall-Levin M, White J, Sanford EM, An P, Sun J, Juhn F, Brennan K, et al. Development and validation of a clinical cancer genomic profiling test based on massively parallel DNA sequencing. Nature biotechnology. 2013; 31: 1023-31. doi: 10.1038/nbt.2696.
4. Liu X, Jian X, Boerwinkle E. dbNSFP: a lightweight database of human nonsynonymous SNPs and their functional predictions. Hum Mutat. 2011; 32: 894-9. doi: 10.1002/humu.21517.
5. Liu X, Jian X, Boerwinkle E. dbNSFP v2.0: a database of human non-synonymous SNVs and their functional predictions and annotations. Hum Mutat. 2013; 34: E2393-402. doi: 10.1002/humu.22376.
6. Harding B, Lemos MC, Reed AA, Walls GV, Jeyabalan J, Bowl MR, Tateossian H, Sullivan N, Hough T, Fraser WD, Ansorge O, Cheeseman MT, Thakker RV. Multiple endocrine neoplasia type 1 knockout mice develop parathyroid, pancreatic, pituitary and adrenal tumours with hypercalcaemia, hypophosphataemia and hypercorticosteronaemia. Endocr Relat Cancer. 2009; 16: 1313-27. doi: 10.1677/ERC-09-0082.

7. Ordog T, Redelman D, Miller LJ, Horvath VJ, Zhong Q, Almeida-Porada G, Zanjani ED, Horowitz B, Sanders KM. Purification of interstitial cells of Cajal by fluorescence-activated cell sorting. Am J Physiol Cell Physiol. 2004; 286: C448-56. doi: 10.1152/ajpcell.00273.2003.
8. Sicklick JK, Li YX, Choi SS, Qi Y, Chen W, Bustamante M, Huang J, Zdanowicz M, Camp T, Torbenson MS, Rojkind M, Diehl AM. Role for hedgehog signaling in hepatic stellate cell activation and viability. Lab Invest. 2005; 85: 1368-80. doi: 10.1038/labinvest.3700349.
9. Sicklick JK, Li YX, Melhem A, Schmelzer E, Zdanowicz M, Huang J, Caballero M, Fair JH, Ludlow JW, McClelland RE, Reid LM, Diehl AM. Hedgehog signaling maintains resident hepatic progenitors throughout life. Am J Physiol Gastrointest Liver Physiol. 2006; 290: G859-70. doi: 10.1152/ajpgi.00456.2005.
10. Sicklick JK, Li YX, Jayaraman A, Kannangai R, Qi Y, Vivekanandan P, Ludlow JW, Owzar K, Chen W, Torbenson MS, Diehl AM. Dysregulation of the Hedgehog pathway in human hepatocarcinogenesis. Carcinogenesis. 2006; 27: 748-57. doi: 10.1093/carcin/bgi292.
11. Rajurkar M, Huang H, Cotton JL, Brooks JK, Sicklick J, McMahon AP, Mao J. Distinct cellular origin and genetic requirement of Hedgehog-Gli in postnatal rhabdomyosarcoma genesis. Oncogene. 2014; 33: 5370-8. doi: 10.1038/onc.2013.480.
12. Ostrowski J, Polkowski M, Paziewska A, Skrzypczak M, Goryca K, Rubel T, Kokoszynska K, Rutkowski P, Nowecki ZI, Vel Dobosz AJ, Jarosz D, Ruka W, Wyrwicz LS. Functional features of gene expression profiles differentiating gastrointestinal stromal tumours according to KIT mutations and expression. BMC Cancer. 2009; 9: 413. doi: 10.1186/1471-2407-9-413.
13. Chen H, Ordog T, Chen J, Young DL, Bardsley MR, Redelman D, Ward SM, Sanders KM. Differential gene expression in functional classes of interstitial cells of Cajal in

murine small intestine. Physiol Genomics. 2007; 31: 492-509. doi: 10.1152/physiolgenomics.00113.2007.

14. Chen H, Redelman D, Ro S, Ward SM, Ordog T, Sanders KM. Selective labeling and isolation of functional classes of interstitial cells of Cajal of human and murine small intestine. Am J Physiol Cell Physiol. 2007; 292: C497-507. doi: 10.1152/ajpcell.00147.2006.

15. Peri LE, Sanders KM, Mutafova-Yambolieva VN. Differential expression of genes related to purinergic signaling in smooth muscle cells, PDGFRalpha-positive cells, and interstitial cells of Cajal in the murine colon. Neurogastroenterol Motil. 2013; 25: e609-20. doi: 10.1111/nmo.12174.

16. Daniel EE, Eteraf T, Sommer B, Cho WJ, Elyazbi A. The role of caveolae and caveolin 1 in calcium handling in pacing and contraction of mouse intestine. J Cell Mol Med. 2009; 13: 352-64. doi: 10.1111/j.1582-4934.2008.00667.x.

17. Wang Y, Marino-Enriquez A, Bennett RR, Zhu M, Shen Y, Eilers G, Lee JC, Henze J, Fletcher BS, Gu Z, Fox EA, Antonescu CR, Fletcher CD, et al. Dystrophin is a tumor suppressor in human cancers with myogenic programs. Nat Genet. 2014. doi: 10.1038/ng.2974.

18. Klein S, Seidler B, Kettenberger A, Sibaev A, Rohn M, Feil R, Allescher HD, Vanderwinden JM, Hofmann F, Schemann M, Rad R, Storr MA, Schmid RM, et al. Interstitial cells of Cajal integrate excitatory and inhibitory neurotransmission with intestinal slow-wave activity. Nat Commun. 2013; 4: 1630. doi: 10.1038/ncomms2626.

19. Chi P, Chen Y, Zhang L, Guo X, Wongvipat J, Shamu T, Fletcher JA, Dewell S, Maki RG, Zheng D, Antonescu CR, Allis CD, Sawyers CL. ETV1 is a lineage survival factor that cooperates with KIT in gastrointestinal stromal tumours. Nature. 2010; 467: 849-53. doi: 10.1038/nature09409.

20. Ward SM, Ordog T, Koh SD, Baker SA, Jun JY, Amberg G, Monaghan K, Sanders KM. Pacemaking in interstitial cells of Cajal depends upon calcium handling by endoplasmic reticulum and mitochondria. J Physiol. 2000; 525 Pt 2: 355-61. doi:
21. Means SA, Cheng LK. Mitochondrial Calcium Handling within the Interstitial Cells of Cajal. Am J Physiol Gastrointest Liver Physiol. 2014. doi: 10.1152/ajpgi.00380.2013.
22. Suzuki H, Takano H, Yamamoto Y, Komuro T, Saito M, Kato K, Mikoshiba K. Properties of gastric smooth muscles obtained from mice which lack inositol trisphosphate receptor. J Physiol. 2000; 525 Pt 1: 105-11. doi:
23. Southwell BR. Localization of protein kinase C theta immunoreactivity to interstitial cells of Cajal in guinea-pig gastrointestinal tract. Neurogastroenterol Motil. 2003; 15: 139-47. doi:
24. Poole DP, Van Nguyen T, Kawai M, Furness JB. Protein kinases expressed by interstitial cells of Cajal. Histochem Cell Biol. 2004; 121: 21-30. doi: 10.1007/s00418-003-0602-8.
25. Wouters M, De Laet A, Donck LV, Delpire E, van Bogaert PP, Timmermans JP, de Kerchove d'Exaerde A, Smans K, Vanderwinden JM. Subtractive hybridization unravels a role for the ion cotransporter NKCC1 in the murine intestinal pacemaker. Am J Physiol Gastrointest Liver Physiol. 2006; 290: G1219-27. doi: 10.1152/ajpgi.00032.2005.
26. Kim BJ, So I, Kim KW. The relationship of TRP channels to the pacemaker activity of interstitial cells of Cajal in the gastrointestinal tract. J Smooth Muscle Res. 2006; 42: 1-7. doi:
27. Gautier L, Cope L, Bolstad BM, Irizarry RA. affy--analysis of Affymetrix GeneChip data at the probe level. Bioinformatics. 2004; 20: 307-15. doi: 10.1093/bioinformatics/btg405.
28. Gomez-Pinilla PJ, Gibbons SJ, Bardsley MR, Lorincz A, Pozo MJ, Pasricha PJ, Van de Rijn M, West RB, Sarr MG, Kendrick ML, Cima RR, Dozois EJ, Larson DW, et al. Ano1 is a selective marker of interstitial cells of Cajal in the human and mouse gastrointestinal

tract. Am J Physiol Gastrointest Liver Physiol. 2009; 296: G1370-81. doi: 10.1152/ajpgi.00074.2009.
