# Supplementary material for: Hedgehog pathway dysregulation contributes to the pathogenesis of human gastrointestinal stromal tumors via GLI-mediated activation of KIT expression
Source: Oncotarget. 2016 Oct 25;7(48):78226–41. doi: 10.18632/oncotarget.12909 (PMC5346634; doi:10.18632/oncotarget.12909)
Supplement: Supplementary file 2 [file oncotarget-07-78226-s002.pdf]

Hedgehog pathway dysregulation contributes to the pathogenesis of human gastrointestinal stromal tumors *via* GLI-mediated activation of *KIT* expression

SUPPLEMENTARY TABLES S1-S8

Authors:
Chih-Min Tang[1]*, Tracy E. Lee[1]*, Sabriya A. Syed[2,3]*, Adam M. Burgoyne[4], Stephanie Y. Leonard[1], Fei Gao[2], Jonathan C. Chan[1], Eileen Shi[5], Juliann Chmielecki[6], Deborah Morosini[6], Kai Wang[6], Jeffrey S. Ross[6], Michael L. Kendrick[7], Michael R. Bardsley[3], Martina De Siena[1], Junhao Mao[8], Olivier Harismendy[9], Tamas Ordog[2,10]*, Jason K. Sicklick[1,5]*

Affiliations:
[1] Division of Surgical Oncology, Department of Surgery, Moores UCSD Cancer Center, University of California, San Diego, La Jolla, California
[2] Enteric Neuroscience Program, Department of Physiology and Biomedical Engineering and Gastroenterology Research Unit, Division of Gastroenterology and Hepatology, Mayo Clinic, Rochester, Minnesota
[3] Department of Biochemistry and Molecular Biology, Mayo Clinic, Rochester, Minnesota
[4] Division of Hematology/Oncology, Department of Medicine, Moores UCSD Cancer Center, University of California, San Diego, La Jolla, California
[5] School of Medicine, University of California, San Diego, La Jolla, California
[6] Foundation Medicine, Inc., Cambridge, Massachusetts
[7] Department of Surgery, Mayo Clinic, Rochester, Minnesota
[8] Department of Molecular, Cell and Cancer Biology, University of Massachusetts, Worchester, Massachusetts
[9] Division of Biomedical Informatics, Moores UCSD Cancer Center, University of California San Diego, La Jolla, CA
[10] Center for Individualized Medicine, Mayo Clinic, Rochester, Minnesota

*These authors contributed equally to this work.

Corresponding Authors:
Jason K. Sicklick, MD, Division of Surgical Oncology, Moores UCSD Cancer Center, University of California, San Diego, 3855 Health Sciences Drive, MC 0987, La Jolla, CA 92093-0987; Tel: 858-822-6173; Fax: 858-228-5153; Email: jsicklick@ucsd.edu

Tamas Ordog, MD, Guggenheim 10, Mayo Clinic, 200 1st Street SW, Rochester, MN 55905; Tel: 507-538-3906; Fax: 507-255-6318; E-mail: ordog.tamas@mayo.edu

Supplementary Table S1. Representation of the "Development_Hedgehog Signaling" network (MetaCore™) in FACS-purified human gastric ICC, mouse small intestinal ICC and mouse gastric ICC-SC.

Cells[a]	Species	Rank[b]	*P*[c]	FDR[c]	Ratio[d]	
					Genes in set	Genes in network
gastric ICC	*Homo sapiens*	29	1.046E-02	0.0566	139	254
ICC-MY	*Mus musculus*	14	1.009E-03	0.0113	34	254
ICC-DMP	*Mus musculus*	9	3.192E-06	0.0000560	46	254
2xSCS2F10	*Mus musculus*	47	7.910E-05	0.000265	105	254

[a], FACS-purified and cultured cells analyzed by Affymetrix Human Genome U133 Plus 2.0 and Mouse Genome 430 2.0 microarrays: human gastric ICC purified by FACS (n=6) and compared to dissociated, unfractionated gastric *tunica muscularis* source tissue samples (n=4) (see details in main text); FACS-purified ICC-MY, mouse small intestinal ICC associated with the myenteric plexus (n=3) and ICC-DMP, mouse small intestinal ICC associated with the deep muscular plexus (n=3) compared to unfractionated BALB/c small intestinal *tunica muscularis* tissues (n=2) [1]; 2xSCS2F10, mouse gastric ICC-SC line (n=3) compared to unfractionated C57BL/6J gastric *tunica muscularis* tissues (n=2) [2]. [b], Ranking of the "Development_Hedgehog Signaling" network among biological process networks detected by MetaCore™ analysis of gene sets with significantly higher expression than controls ($\log_2$ fold change >1, Benjamini-Hochberg Q<0.05). See top 50 process networks for each cell type in **Supplementary Table S8**. [c], P values and false discovery rates (FDR) from the network analysis. [d], Ratio of differentially expressed genes and genes in the "Development_Hedgehog Signaling" network.

Supplementary Table S2. Sources of human GIST microarray data.

**NCBI GEO[a] Series: GSE17743** [3][b]		**NCBI GEO Series: GSE8167** [4]		**NCBI GEO Series: GSE20708** [5]	
**Label[c]**	**Sample ID**	**Label**	**Sample ID**	**Label**	**Sample ID**
GIST_KIT_Ex11_1	GSM442936	GIST_WT_8	GSM202198	GIST_KIT_Ex11_15	GSM519700
GIST_KIT_Ex11_2	GSM442937	GIST_WT_9	GSM202199	GIST_KIT_Ex9_2	GSM519701
GIST_KIT_Ex11_3	GSM442938	GIST_KIT_Ex11_22	GSM202200	GIST_PDGFRA_Ex12_3	GSM519702
GIST_PDGFRA_Ex18_1	GSM442939	GIST_KIT_Ex11_23	GSM202204	GIST_WT_4	GSM519703
GIST_KIT_Ex11_4	GSM442940	GIST_KIT_Ex11_24	GSM202205	GIST_KIT_Ex11_16	GSM519704
GIST_KIT_Ex11_5	GSM442941	GIST_KIT_Ex11_25	GSM202206	GIST_KIT_Ex11_17	GSM519705
GIST_KIT_Ex9_1	GSM442942	GIST_WT_10	GSM202207	GIST_WT_5	GSM519706
GIST_WT_1	GSM442943	GIST_KIT_Ex11_26	GSM202208	GIST_KIT_Ex11_18	GSM519707
GIST_WT_2	GSM442944	GIST_WT_11	GSM202209	GIST_PDGFRA_Ex14_1	GSM519708
GIST_KIT_Ex11_6	GSM442945	GIST_KIT_Ex11_27	GSM202210	GIST_KIT_Ex11_19	GSM519710
GIST_KIT_Ex11_7	GSM442946	GIST_KIT_Ex11_28	GSM202211	GIST_PDGFRA_Ex18_10	GSM519711
GIST_PDGFRA_Ex18_2	GSM442947	GIST_KIT_Ex11_29	GSM202212	GIST_KIT_Ex11_20	GSM519712
GIST_PDGFRA_Ex12_1	GSM442948	GIST_KIT_Ex11_30	GSM202213	GIST_WT_6	GSM519715
GIST_PDGFRA_Ex18_3	GSM442949	GIST_KIT_Ex11_31	GSM202216	GIST_PDGFRA_Ex18_11	GSM519716
GIST_PDGFRA_Ex18_4	GSM442950	GIST_KIT_Ex11_32	GSM202217	GIST_KIT_Ex11_21	GSM519717
GIST_WT_3	GSM442951	GIST_KIT_Ex11_33	GSM202218	GIST_WT_7	GSM519718
GIST_PDGFRA_Ex18_5	GSM442952	GIST_KIT_Ex11_34	GSM202220	GIST_PDGFRA_Ex12_4	GSM519720
GIST_PDGFRA_Ex12_2	GSM442953	GIST_KIT_Ex11_35	GSM202221		
GIST_KIT_Ex11_8	GSM442954	GIST_WT_12	GSM202223		
GIST_PDGFRA_Ex18_6	GSM442955	GIST_WT_13	GSM202225		
GIST_PDGFRA_Ex18_7	GSM442956	GIST_KIT_Ex11_36	GSM202226		
GIST_KIT_Ex11_9	GSM442957	GIST_WT_14	GSM202227		
GIST_KIT_Ex11_10	GSM442958	GIST_KIT_Ex11_37	GSM202228		
GIST_KIT_Ex11_11	GSM442959				
GIST_KIT_Ex11_12	GSM442960				
GIST_PDGFRA_Ex18_8	GSM442961				
GIST_KIT_Ex11_13	GSM442962				
GIST_PDGFRA_Ex18_9	GSM442963				
GIST_KIT_Ex11_14	GSM442964				

[a], NCBI GEO, National Center for Biotechnology Information Gene Expression Omnibus.

[b], References:

1. Chen H, Ordog T, Chen J, Young DL, Bardsley MR, Redelman D, Ward SM, Sanders KM. Differential gene expression in functional classes of interstitial cells of Cajal in murine small intestine. Physiol Genomics. 2007; 31: 492-509. doi: 10.1152/physiolgenomics.00113.2007.
2. Dave M, Hayashi Y, Gajdos GB, Smyrk TC, Svingen PA, Kvasha SM, Lorincz A, Dong H, Faubion WA, Jr., Ordog T. Stem cells for murine interstitial cells of cajal suppress cellular immunity and colitis via prostaglandin E2 secretion. Gastroenterology. 2015; 148: 978-90. doi: 10.1053/j.gastro.2015.01.036.
3. Ostrowski J, Polkowski M, Paziewska A, Skrzypczak M, Goryca K, Rubel T, Kokoszynska K, Rutkowski P, Nowecki ZI, Vel Dobosz AJ, Jarosz D, Ruka W, Wyrwicz LS. Functional features of gene expression profiles differentiating gastrointestinal stromal tumours according to KIT mutations and expression. BMC Cancer. 2009; 9: 413. doi: 10.1186/1471-2407-9-413.
4. Yamaguchi U, Nakayama R, Honda K, Ichikawa H, Hasegawa T, Shitashige M, Ono M, Shoji A, Sakuma T, Kuwabara H, Shimada Y, Sasako M, Shimoda T, et al. Distinct gene expression-defined classes of gastrointestinal stromal tumor. J Clin Oncol. 2008; 26: 4100-8. doi: 10.1200/JCO.2007.14.2331.
5. Astolfi A, Nannini M, Pantaleo MA, Di Battista M, Heinrich MC, Santini D, Catena F, Corless CL, Maleddu A, Saponara M, Lolli C, Di Scioscio V, Formica S, et al. A molecular portrait of gastrointestinal stromal tumors: an integrative analysis of gene expression profiling and high-resolution genomic copy number. Lab Invest. 2010; 90: 1285-94. doi: 10.1038/labinvest.2010.110.

[c], Labels used in **Figure 3A**. Mutated gene, exon number and serial number are indicated.

Supplementary Table S3. Antibodies and other reagents used for the isolation of ICC, *NOT* ICC and hematopoietic cells from human gastric *tunica muscularis.*

Target	Supplier	Host/Source	Clone/ID	Isotype	Label	Final conc. or amount/10^6 cells/100 µL
KIT[a]	BioLegend	Mouse mc[b]	104D2	IgG_1, κ	APC[c]	0.5 µg
CD34[d]	BD Pharmingen	Mouse mc	581	IgG_1, κ	PE[e]	0.0625 µg
FCER1A[f]	BioLegend	Mouse mc	AER-37	IgG_{2b}, κ	PE-Cy7[g]	0.25 µg
CD11B[h]	BD Pharmingen	Mouse mc	ICRF44	IgG_1, κ	PE-Cy7	0.25 µg
CD45[i]	BD Pharmingen	Mouse mc	HI30	IgG_1, κ	PE-Cy7	0.1 µg
Dead cells[j]	eBioscience		Fixable Viability Dye		APC-eFluor 780	1 µL/mL
Fcγ receptors	BD Pharmingen		Purified NA/LE Human BD Fc Block™			20 µL

[a], v-kit Hardy-Zuckerman 4 feline sarcoma viral oncogene homolog (CD117); [b], mc, monoclonal; [c], APC, allophycocyanin; [d], CD34 molecule; [e], PE, phycoerythrin; [f], FCER1A, Fc fragment of IgE, high affinity I, receptor for; alpha polypeptide; [g], Cy7, cyanine 7; [h], CD11B, integrin, alpha M (complement component 3 receptor 3 subunit) (ITGAM, MAC-1); [i], CD45, protein tyrosine phosphatase, receptor type, C (PTPRC), alias: leukocyte common antigen; [j], Fixable Viability Dye. Suppliers: BioLegend, Inc., San Diego, CA; BD Pharmingen, Becton, Dickinson and Company, Franklin Lakes, NJ; eBioscience, Inc., San Diego, CA.

Supplementary Table S4. Configuration of the Becton Dickinson LSR II flow cytometer.

Laser	Excitation wavelength (nm)	Dichroic filter (nm)	Emission filter (nm; peak/bandwidth)	Detector type	Light scatter or fluorochrome used
Coherent® Sapphire™ 20 mW	488			Photodiode	Forward scatter
			488/10	PMT[a]	Side scatter
		505 LP[b]	530/30	PMT	Unused
		550 LP	575/26	PMT	PE[c]
		595 LP	610/20	PMT	Unused
		685 LP	695/40	PMT	Unused
		735 LP	780/60	PMT	PE-Cy7[d]
Coherent® CUBE 100 mW	407		450/50	PMT	Unused
		505LP	525/50	PMT	Unused
		535 LP	590/40	PMT	Unused
		595 LP	610/20	PMT	Unused
		630 LP	670/30	PMT	Unused
		670 LP	710/50	PMT	Unused
Coherent® CUBE 40 mW	640		660/20	PMT	APC[e]
		685 LP	712/20	PMT	Unused
		735 LP	780/60	PMT	APC-eFluor 780

[a], PMT, photomultiplier tube; [b], LP, long-pass; [c], PE, phycoerythrin; [d], Cy7, cyanine 7; [e], APC, allophycocyanin.

Supplementary Table S5. Patient, tissue and harvested cell information.

Age (yr)	Sex	Surgery	Sample	ICC count[a]	*NOT* ICC count[b]	HP^+ count[c]
33	M	Duodenal switch	ICC_1	1,009		
32[d]	F	Sleeve gastrectomy	ICC_2	5,528		
23[d]	F	Sleeve gastrectomy				
60	F	Sleeve gastrectomy	ICC_3	4,929		
51	F	Sleeve gastrectomy	ICC_4	10,448		
52	M	Sleeve gastrectomy	ICC_5	4,584		
51	F	Sleeve gastrectomy	NOT_ICC		428,676	
31	F	Sleeve gastrectomy	TISSUE_1			
51	M	Sleeve gastrectomy	TISSUE_2			
51	F	Sleeve gastrectomy	TISSUE_3			
23	F	Sleeve gastrectomy	TISSUE_4			
57	F	Sleeve gastrectomy	HP_1			127,186
47	F	Sleeve gastrectomy	ICC_6	2,541		
			HP_2			397,368
27	M	Sleeve gastrectomy	HP_3			552,154

[a], ICC, interstitial cells of Cajal: KIT^+CD34^- cells excluding the Fixable Viability Dye allophycocyanin (APC)-eFluor 780 (live cells) and not labeled with antibodies against the hematopoietic markers CD45, FCER1A, and anti-CD11B (HP^-); [b], *NOT* ICC: live, $HP^-KIT^-CD34^-$ cells; [c], HP^+: live HP^+ cells. [d], Samples combined for processing due to small size.

Supplementary Table S6. RT-PCR primers for human samples.

Gene	Direction	Sequence	Amplicon size (bp)
SHH	Forward	GCTCGGTGAAAGCAGAGAAC	109
	Reverse	CTCAGGTCCTTCACCAGCTT	
IHH	Forward	TCCGTCAAGTCCGAGCAC	109
	Reverse	GCCTCACGGCTGACAAGG	
PTCH1	Forward	CCACCAGACGCTGTTTAGTCA	72
	Reverse	CGATGGAGTCCTTGCCTACAA	
SMO	Forward	CAGTTCCAAACATGGCAAACAG	200
	Reverse	TGCTATGTCAGGCCAATGTGA	
GLI1	Forward	TGCAGTAAAGCCTTCAGCAATG	132
	Reverse	TTTTCGCAGCGAGCTAGGAT	
GLI2	Forward	CGAGAAACCCTACATCTGCAAGA	88
	Reverse	GTGGACCGTTTTCACATGCTT	
GLI3	Forward	AAACCCCAATCATGGACTCAAC	98
	Reverse	TACGTGCTCCATCCATTTGGT	
KIT	Forward	TGTCATCAGCCACCATCCTA	156
	Reverse	GCAGAATTGGAGAAGCCTTG	
KIT[a]	Forward	GGGATTTTCTCTGCGTTCTG	99
	Reverse	GATGGATGGATGGTGGAGAC	
ANO1	Forward	TGTCAGAGCCAAAGACATCG	183
	Reverse	AAGGAGGAGAGGGTGTGGTT	
ACTB	Forward	AATGTGGCCGAGGACTTTGATTGC	93
	Reverse	AGGATGGCAAGGGACTTCCTGTAA	

[a], Used for microarray validation.

Supplementary Table S7. RT-PCR primers for mouse samples.

Gene	Direction	Sequence	Amplicon size (bp)
Shh	Forward	CTGGCCAGATGTTTTCTGGT	117
	Reverse	TAAAGGGGTCAGCTTTTTGG	
Ihh	Forward	CCGAACCTTCATCTTGGTG	124
	Reverse	ACAGATGGAATGCGTGTGAA	
Ptch1	Forward	ATGCTCCTTTCCTCCTGAAACC	168
	Reverse	TGAACTGGGCAGCTATGAAGTC	
Smo	Forward	GCCTGGTGCTTATTGTGG	75
	Reverse	GGTGGTTGCTCTTGATGG	
Gli1	Forward	AACTCCACAGGCACACAGG	79
	Reverse	GCTCAGGCTTCTCCTCTCTC	
Gli2	Forward	CCATTCATAAGCGGAGCAAG	105
	Reverse	CCAGGTCTTCCTTGAGATCG	
Gli3	Forward	GCTCTTCAGCAAGTGGTTCC	122
	Reverse	CTGTCGGCTTAGGATCTGTTG	
Kit	Forward	GGCTAGCCAGAGACATCAGG	159
	Reverse	AGGAGAAGAGCTCCCAGAGG	
Actb	Forward	GCTCTGGCTCCTAGCACC	74
	Reverse	CCACCGATCCACACAGAGTACTTG	

Supplementary Table S8. Top 50 process networks for each cell type (in attached MS Excel file).
